# Enhanced Energetic Performance via the Combination of Furoxan and Oxa-[5,5]bicyclic Structures

**DOI:** 10.3390/ijms24108846

**Published:** 2023-05-16

**Authors:** Qi Zhang, Xun Zhang, Siping Pang, Chunlin He

**Affiliations:** 1 School of Materials Science & Engineering, Beijing Institute of Technology, Beijing 100081, China; zhangq135@bit.edu.cn (Q.Z.); zhangxun@bit.edu.cn (X.Z.); 2 Experimental Center of Advanced Materials, School of Materials Science & Engineering, Beijing Institute of Technology, Beijing 100081, China; 3 Chongqing Innovation Center, Beijing Institute of Technology, Chongqing 401120, China

**Keywords:** energetic compounds, oxygen heterocycles, furoxans, [5,5]-fused rings

## Abstract

Three new compounds based on the combination of furoxan (1,2,5-oxadiazole *N*-oxide) and oxa-[5,5]bicyclic ring were synthesized. Among them, the nitro compound showed satisfactory detonation properties (*Dv*, 8565 m s^−1^; *P*, 31.9 GPa), which is comparable to the performance of RDX (a classic high-energy secondary explosive). Additionally, the introduction of the *N*-oxide moiety and oxidation of the amino group more effectively improved the oxygen balance and density (*d*, 1.81 g cm^−3^; OB%, +2.8%) of the compounds compared to furazan analogues. Combined with good density and oxygen balance as well as moderate sensitivity, this type of furoxan and oxa-[5,5]bicyclic structure will open up a platform for the synthesis and design of new high-energy materials.

## 1. Introduction

**Figure 1:**
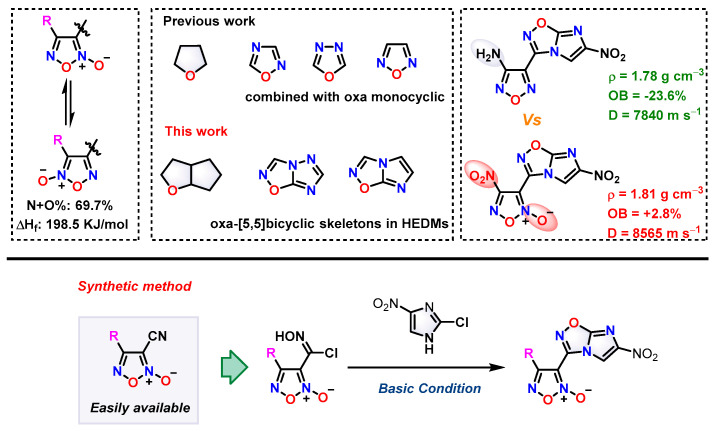
Furoxan and oxa-[5,5]bicyclic skeleton in energetic compounds.

Furoxan is a typical planar nitrogen heterocyclic skeleton containing *N*-oxide units [1,2,3,4]. It is commonly used as structural block of high-energy materials due to its “latent nitro” in the ring [5,6]. Benefitting from the dense molecular structure and high nitrogen and oxygen content (69.7%), furoxan could effectively improve the oxygen balance (OB%) and provide higher energy density and prominent enthalpy of formation (198.5 kJ mol^−1^) as an explosive group for energetic compounds [7]. The furoxan heterocyclic structure has the form of 2-oxide and 5-oxide tautomers, which can undergo tautomerism through the active *cis*-1,2-dinitrosoethylene intermediate under heating conditions. The existence of tautomers reduces the stability of furoxan skeleton and also causes difficulties in energetic synthesis (Figure 1) [8]. Owing to the good detonation properties, furoxan with attached heterocyclic energetic compounds has been extensively studied in the past decades [9,10]. However, the relatively high sensitivity and poor thermal stability remain constraints on its applications. Common energetic compounds containing furoxan include structures that multiple furoxans directly connect or through the NHCH_2_NH, N=N (O) bridge connection [11,12,13]. Another kind of structure is mainly based on the combination of the furoxan ring and five-membered heterocycles, such as furoxan connected with isoxazole [14], 1,2,4-oxadiazole [15,16], 1,3,4-oxadiazole [17,18], 1,2,5-oxadiazole [19,20], 1,2,4-triazole [21,22], tetrazole [23,24], etc. (Figure 1). Nevertheless, the synthetic reaction pattern of these compounds is relatively fixed and unitary, generally through the ring-closing reaction of amino-oxime, amino-hydrazone, and hydrazide furoxan with cyanogen bromide. Although successive progress has been made on the direct connection of furoxan with the five-membered ring, a structure connected with the bicyclic structure has not been reported so far.

Recent studies have shown that the introduction of a fused-ring skeleton can effectively increase the enthalpy of formation, thereby further enhancing detonation performance [25,26,27]. In order to simultaneously accomplish the oxygen balance boost, the oxa-[5,5]bicyclic ring is a preeminent candidate structure. Due to the high tension of the skeleton ring, the oxa-[5,5]bicyclic structure can store more chemical energy, giving it great application potential in the area of high-energy-density materials (HEDMs) [28]. Oxa-[5,5]bicyclic rings have a variety of atomic arrangements and may form a large number of different skeleton combinations. Although this class of structure has received more attention in recent years, only four kinds of skeleton structures have been applied in energetic compounds so far. The number of neutral energetic compounds is few, and we lack knowledge of their physical and chemical properties. In 2017, Tang et al. prepared pyrazolo [3,4-c]furazan *N*-oxide and several nitrogen-rich energetic salts that exhibited excellent detonation properties but had high impact and were friction-sensitive [29]. In 2020, 4*H*-[1,2,3]triazolo [4,5-*c*][1,2,5]oxadiazole 5-oxide and its salts, with moderate impact and friction sensitivity, were reported by Churakov [30]. Until 2022, our group has worked on constructing two new oxygen-containing [5,5]-fused skeletons with more modifiable sites, namely the [1,2,4]triazolo [1,5-d][1,2,4]oxadiazole and imidazo [1,2-d][1,2,4]oxadiazole ring system (Figure 1) [31,32]. However, attributing to the instability of N-O and C-N bonds in the oxa-[5,5]bicyclic ring, the two rings may have simultaneous cracking. Meanwhile, due to the limited synthesis methods and the difficulty of energetic derivation, the type and quantity of energetic compounds with the oxa-[5,5]bicyclic structure actually synthesized are very limited.

Based on our previous work, we propose starting from easily available and obtained 4-amino-3-cyano-1,2,5-oxadiazole 2-oxide to realize the synthesis of furoxan connected with oxa-[5,5]bicyclic ring energetic compounds, through conventional cyano functional group conversion. The introduction of the *N*-oxide moiety and amino oxidation can further improve the oxygen balance and energy density compared to furazan analogues (Figure 1) [33]. Considering that furoxan is relatively sensitive to alkaline conditions and high temperature, there will be problems regarding the stability of target products and the competitive reaction of chloroxime condensation under alkaline conditions in the reaction process. In this work, the oxa-[5,5]bicyclic ring energetic compounds of furoxan were obtained through the ring-closing reaction of amino-protected chloroxime furoxan and 2-chloro-4-nitro-1*H*-imidazole under alkaline conditions, followed by deprotection of the amino group. The oxidation products and azo products were also successfully prepared, and all compounds were deeply characterized using ^1^H, ^13^C, ^14^N NMR spectroscopy, high-resolution mass spectrometry (HRMS), infrared (IR), and elemental analysis. Among them, amino and nitro structures were clearly identified by X-ray single-crystal diffraction analysis. Overall, this new cyclization mode could construct furoxan and oxa-[5,5]bicyclic structures to enrich their types of energetic compounds by conducting cyclization reaction between furoxan containing a chloroxime group and N-α halogenated azole compounds.

## 2. Results and Discussion

**Scheme 1:**
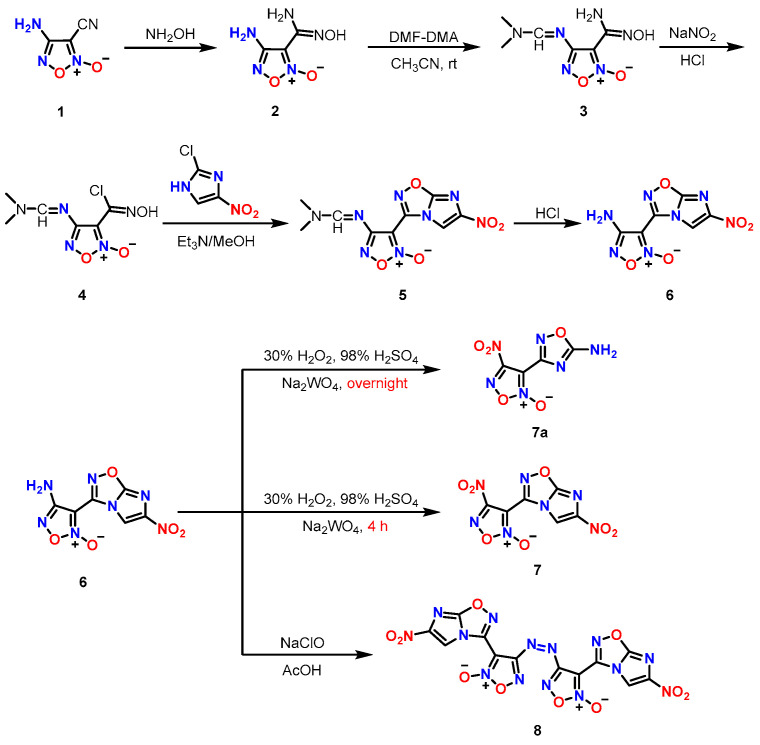
Synthetic routes for preparing compounds **6**–**8**.

In Scheme 1, compound **1** was synthesized in good yield by oxidation of *o*-dioxime by (diacetoxyiodo)benzene [33]. Compound **4** was prepared according to the literature [34]. Then, a methanol suspension of **4** and 1.0 equivalent of 2-chloro-4-nitro-1*H*-imidazole with 2.0 eq triethylamine was stirred overnight at room temperature to afford target product **5** in 75% yield. In this process, 3,4-bis(4′-dimethylaminomethyleneaminofuroxano-3′)furoxan can be inevitably observed as the byproduct. Then, the amino group in compound **5** can easily split the amidine protection by reacting with the hydrochloric acid aqueous solution to give the amino compound **6**. A mixture of 30% H_2_O_2_, Na_2_WO_4_, and H_2_SO_4_ was able to realize the oxidation of the amino group to obtain nitro product **7** in 82% yield. Notably, if the reaction time is prolonged to overnight, the [5,5]-fused ring will break to give compound **7a** instead of **7**, so the reaction time was controlled to 4 h. The structures of **5**–**7** and **7a** were clarified by single-crystal X-ray diffraction (**CCDC** 2237350, 2237351, 2237352, and 2253262; Tables S1–S4). To construct an intramolecular azo bridge compound **8**, oxidants trichloroisocyanuric acid (TCICA) and 10% sodium hypochlorite aqueous solution were tested. It was found that when TCICA was the oxidant, the target product could be obtained with a yield of 62%, but the stability of the product in the reaction system was relatively poor, so it required quenching immediately after the reaction finished. In addition, the target compound was difficult to separate and purify due to the presence of impurities in the reaction system. The target product can be observed in the oxidation system of 10% NaClO solution and sodium bicarbonate, but it decomposes quickly. Therefore, the combination of 10% NaClO solution and acetic acid was chosen as the most appropriate reaction conditions, and compound **8** was smoothly prepared with 79% yield.

**Scheme 2:**
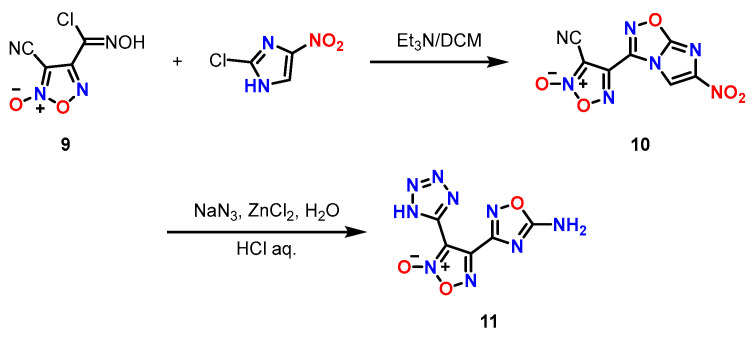
Synthetic routes of compounds **10**–**11**.

We also tried to use 4-(chloro(hydroxyimino)methyl)-3-cyano-1,2,5-oxadiazole 2-oxide (**9**) as a raw material to introduce the cyano group for further derivatization (Scheme 2). Under similar reaction conditions, the target product **10** (**CCDC** 2252471; Table S5) can be obtained smoothly, but it is necessary to change the solvent to dichloromethane. Then, compound **10** underwent a cycloaddition reaction with sodium azide, but after acidification to pH 1~2 with an appropriate concentrated hydrochloric, the [5,5]-fused ring was degraded to afford compound **11** (**CCDC** 2252472; Table S6), indicating the instability of the oxa-[5,5]bicyclic structure.

**Scheme 3:**
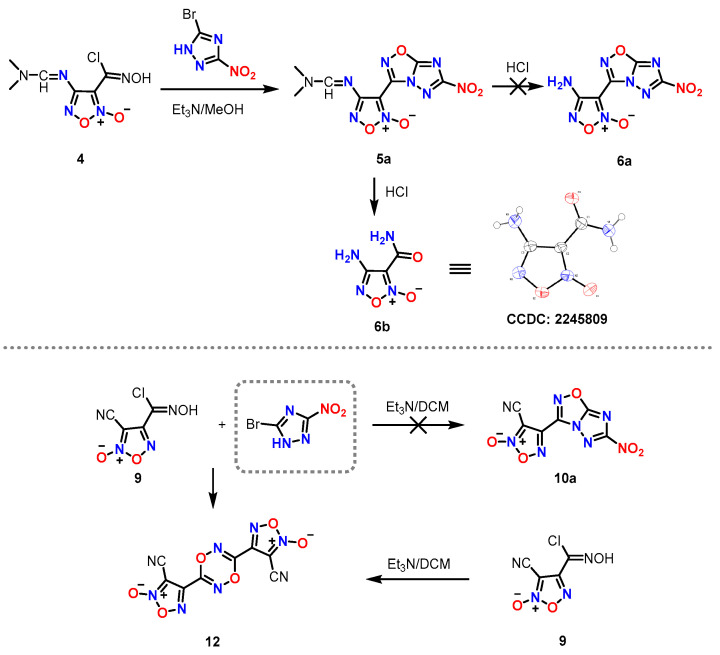
Synthetic procedures for preparing compounds **5a** and **12**.

Considering that 1,2,4-triazole has higher density, enthalpy of formation, and better detonation performance than imidazole, the similar ring-closing reaction of compound **4** with 5-bromo-3-nitro-1*H*-1,2,4-triazole was further attempted (Scheme 3). Fortunately, the target product **5a** was successfully obtained with a yield of 65%, and its structure was confirmed by single-crystal X-ray diffraction (**CCDC** 2245807; Table S7). However, the amino product **6a** could not be obtained smoothly during the next amino deprotection process, and only a small amount of the byproduct 4-amino-3-carbamoyl-1,2,5-oxadiazole 2-oxide **6b** was obtained (**CCDC** 2245809; Table S8). In addition to hydrochloric acid aqueous solution, using other reaction conditions such as trifluoroacetic acid or ZnCl_2_, the target product was not acquired. As for the 4-(chloro(hydroxyimino)methyl)-3-cyano-1,2,5-oxadiazole 2-oxide, unexpectedly, a cyclization reaction occurred to form 1,4,2,5-dioxadiazine bridge-based furoxans instead of the expected reaction with 5-bromo-3-nitro-1*H*-1,2,4-triazole. We conducted a validation experiment, and under the condition of triethylamine, two molecules of compound **9** were cyclized to obtain **12** (**CCDC** 2252474; Table S9).

**Figure 2:**
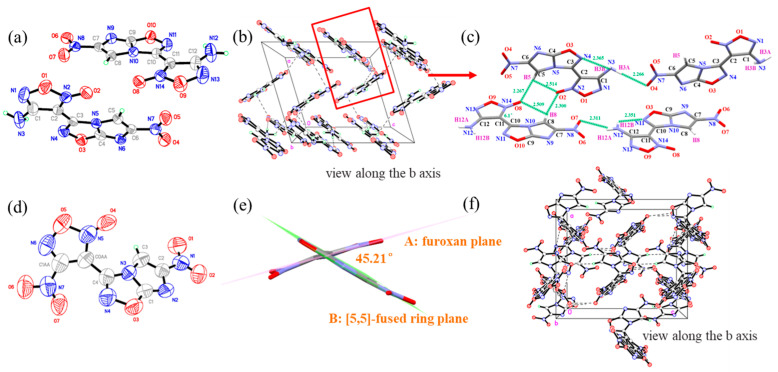
(**a**–**c**) X-ray single-crystal diffraction and crystal packing of compound **6**; (**d**–**f**) X-ray single-crystal diffraction and crystal packing of compound **7**.

Crystals of **6** and **7** suitable for X-ray diffraction were obtained by slow evaporation of ethyl acetate and petroleum ether solutions at room temperature. Compound **6** crystallizes in the monoclinic space group *P*2_1_/*c* with a calculated density of 1.807 g cm^−3^ at 298 K and a unit cell consisting of four molecules (*Z* = 4) (Figure 2a). All atoms in **6** are almost coplanar, with the dihedral angles of N5-C3-C2-C1, N7-C6-C5-N5, and N2-C2-C1-N3 as 177.6°, 179.1° and 176.3°, respectively. Compound **6** shows a wave-like stacking in the packing diagram (Figure 2b). Two typical intramolecular hydrogen bonds [N(3)–H(3B)/N(4) and N(12)–H(12B)/N(11)] and three intermolecular hydrogen bonds [N(3)–H(3A)/O(4)*^a^*, N(3)–H(3B)/O(9)*^b^* and N(12)–H(12A)/O(7)*^c^*, symmetry code *a*: 1 + *x*, *y*, *z*; *b*: −*x*, 1/2 + *y*, 3/2 − *z*; *c*: 1 + *x*, 1/2 − *y*, −1/2 + *z*] were observed in the crystal structure of compound **6** (Figure 2c). Compound **7** crystallized in the orthorhombic space group *Pbca* with eight molecular units (*Z* = 8) in the unit cell and a calculated density of 1.810 g cm^−3^ at 298 K (Figure 2d). After the oxidation of the amino group in compound **6** to a nitro group, the furoxan plane in the structure of compound **7** formed an angle of 45.21° with the plane of [5,5]-fused ring (Figure 2e), and compound **7** exhibited a mixed stacking in the packing diagram (Figure 2f). In addition, with the substitution of the nitro for the amino group, compound **7** lost favorable hydrogen bonding interactions due to the lack of hydrogen atoms, leading to an increase in friction and impact sensitivities.

**Figure 3:**
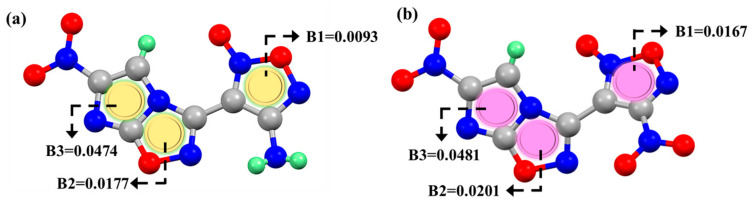
(**a**) The multicenter bond orders of **6**; (**b**) the multicenter bond orders of **7**.

The physical properties of **6**–**8** compared with the properties of RDX are summarized in Table 1. Thermal stability determines the adaptability of energetic materials to practical applications and is one of the important physical properties for evaluating their safety. The thermal stabilities of the new compounds were determined by differential scanning calorimetry (DSC) with a heating rate of 5 °C min^−1^. These compounds exhibited good thermal stability, while azo compound **8** was the least thermally stable compound, decomposing at 158.8 °C. The decomposition temperature (onset) of compound **6** was 163.6 °C, and the thermal stability of the compound **7** was improved, with a decomposition temperature of 170.3 °C. To further investigate the thermal stability of compounds **6** and **7** from the perspective of molecular structure, the multicenter bond orders were calculated by Multiwfn (Figure 3) [35,36]. The results demonstrate that the furoxan ring and oxa-[5,5]bicyclic ring of **7** displays higher multicenter bond orders than that of **6** (**6**: BM = 0.0093/0.0177/0.0474; **7**: BM = 0.0167/0.0201/0.0481), indicating that **7** exhibits stronger aromaticity.

The densities of compounds **6**–**8** were measured using a gas pycnometer at 25 °C, and their experimental densities ranged from 1.78 to 1.82 g cm^−3^. The densities of compounds **6** and **7** were slightly higher than that of classic explosive RDX (1.80 g cm^−3^). The heat of formation (HOF) values of compounds **6**–**8** were calculated by the Gaussian 09 (Revision E.01) program using the isodesmic reactions shown in the ESI (Figure S1, Table S10). The presence of the high enthalpies of furoxan and [5,5]-fused-ring skeletons caused the three prepared compounds to feature high positive HOF, much higher than RDX (70.3 kJ mol^−1^), ranging from 370.7 kJ mol^−1^ to 1212.7 kJ mol^−1^. As shown in Table 1, among these compounds, the azo compound **8** demonstrated the highest positive HOF (1212.7 kJ mol^−1^), which is attributed to the introduction of the azo group adding more high-energy N=N and C-N bonds.

Together with the measured densities, some important energetic performance measurements, such as the energy and temperature of the explosion, were evaluated by using the EXPLO5 6.05 program [37]. Compounds **6**–**8** showed good detonation properties, and compound **7** especially possesses excellent detonation velocity and pressure. The calculated detonation velocity and detonation pressure of nitro product **7** were 8565 m s^−1^ and 31.9 GPa respectively, which are comparable to RDX (*Dv* = 8795 m s^−1^, *P* = 34.9 GPa), elucidating its potential as an RDX replacement candidate. The heat of detonation and detonation temperature were also evaluated and compared with RDX. Similar to other detonation properties, compound **7** has the highest heat of detonation (5812 kJ kg^−1^) and temperature of detonation (4490 K), both higher than that of RDX. Impact and friction sensitivities were recorded by BAM fall-hammer and BAM friction-sensitivity tester. Compounds **6**–**8** have similar moderate sensitivity, as shown in Table 1. Because of the existing furoxan, it is not surprising that these compounds displayed relatively high sensitivity (*IS*: 3~5 J, *FS*: 80~84 N). Additionally, due to the introduction of the oxygen-containing heterocyclic and furoxan skeleton, the nitrogen and oxygen contents of the three compounds were more than 70%, and the oxygen balance of compound **7** was also significantly improved.

**Figure 4:**
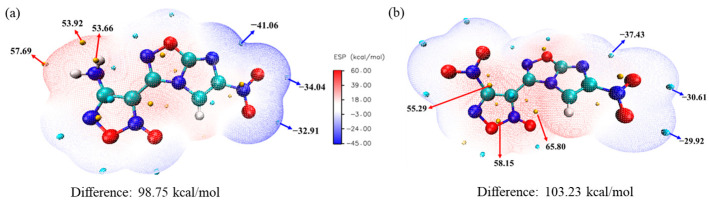
(**a**) Electrostatic potential surfaces (ESP) for compound **6**; (**b**) electrostatic potential surfaces (ESP) for compound **7**. The blue and red spheres in the diagram represent the surface local minima and maximum of ESP, respectively.

The calculated electrostatic potentials (ESP) of the molecular surfaces are closely related to sensitivities [38]. In general, the greater positive ESP values and more extensive electropositive regions (red areas) usually lead to more sensitive energetic compounds. As shown in Figure 4, compound **7** has higher ESP maximum values (+65.80 kcal mol^−1^ for **7**, +57.69 kcal mol^−1^ for **6**) and a larger electrostatic potential difference (103.23 kcal mol^−1^ for **7**, 98.75 kcal mol^−1^ for **6**) compared to compound **6**, which agrees with the experimental results.

**Figure 5:**
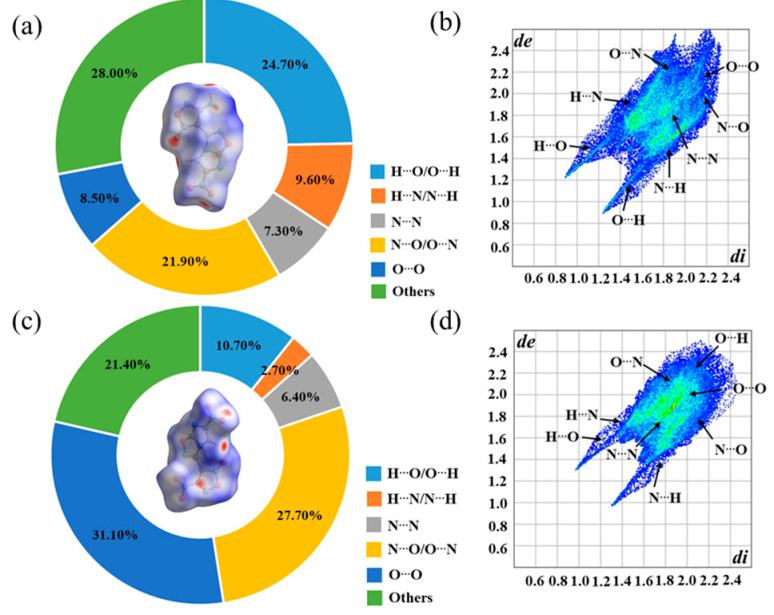
Hirshfeld surfaces (inside) and pie graph showing the percentage contributions of the individual atomic contacts to the Hirshfeld surfaces of **6** (**a**) and **7** (**c**). Fingerprint plots of **6** (**b**) and **7** (**d**).

The two-dimensional (2D) fingerprint and associated Hirshfeld surfaces of compounds **6** and **7** were investigated to gain a further understanding of the intermolecular interactions (Figure 5) [39]. It can be intuitively noted that compound **6** is nearly plate-shaped, with most of the red dots being distributed on the surface edges. More hydrogen bonding interactions (N···H, 9.6%; O···H 24.7%) were observed in compound **6** than **7** (N···H, 2.7%; O···H 10.7%). Meanwhile, the π–π interactions (N···O/O···N, N···N contact) accounted for 29.2% in compound **6** and 34.1% in compound **7**, demonstrating that they also play an important role in reducing the sensitivity of the compounds. Other than the favorable contact, the unfavorable O···O contact of compound **7** is evidently higher than that of compound **6**, which results in a relatively high sensitivity.

**Figure 6:**
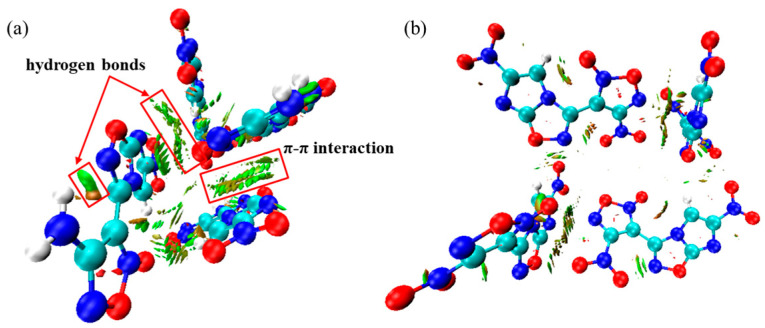
Noncovalent interaction (NCI) analysis for compounds **6** (**a**) and **7** (**b**).

The non-covalent interaction (NCI) plots of compounds **6** and **7** were studied to further investigate their inter- and intra-molecular interactions (Figure 6) [40]. The existence of green isosurfaces in **6** is greater than that in **7**, indicating the presence of extensive π–π and hydrogen bonding interactions for compound **6**. Therefore, it can be inferred from these calculations that compound **6** had a lower sensitivity than **7**.

## 3. Materials and Methods

All reagents were purchased from commercial sources (Energy Chemical (Energy Chemical, Shanghai, China), Adamas-beta^®^ (Titan, Shanghai, China), J&K Scientific (J&K Scientific, Shanghai, China), Sigma-Aldrich (Merck, Shanghai, China)) and used without purification unless otherwise mentioned. The products were purified by column chromatography over silica gel (200–300 size). ^1^H, ^13^C, and ^14^N nuclear magnetic resonance (NMR) spectra were recorded at 25 °C on a Bruker 400 MHz, 100 MHz; TMS was used as internal standard. Infrared spectra (IR) were obtained on a PerkinElmer Spectrum BX FT-IR instrument equipped with an ATR unit at 25 °C. Elemental analyses of C/H/N were investigated on a Vario EL III Analyzer. High-resolution mass spectra (HRMS) were recorded on Thermo Scientific LTQ Orbitrap XL and Thermo Scientific Q Exactive by using ESI method. The onset decomposition temperature was recorded on TA Discovery DSC 25 or METT-FT 900 at a heating rate of 5 °C min^−1^ under a dry nitrogen atmosphere. Impact and friction sensitivities were measured with a BAM fall-hammer and friction tester. Densities were calculated at 298K based on crystal structure data and determined at room temperature by employing a Micromeritics AccuPyc II 1345 gas pycnometer. All quantum chemical calculations were carried out using the Gaussian 09 program package and visualized by GaussView 5.0. The geometric optimization and frequency analyses of the structures were carried out using the B3LYP functional with 6-311 + g(d,p) basis set, and single energy points were calculated at the M062X/6-311g(d,p) level. All of the optimized structures were characterized to be true local energy minima on the potential energy surface without imaginary frequencies.

5-Bromo-3-nitro-1H-1,2,4-triazole

Prepared according to the literature [41]. Yield 89%.

2-Chloro-4-nitro-1H-imidazole

Commercially available reagent.

4-Amino-3-cyano-1,2,5-oxadiazole 2-oxide (1)

Prepared according to the literature [33]. Yield 70%.

3-Chlorohydroximoyl-4-dimethylaminomethyleneaminofuroxan (4)

Prepared according to the literature [34]. Yield 87%.

4-(((Dimethylamino)methylene)amino)-3-(6-nitroimidazo [1,2-d][1,2,4]oxadiazol-3-yl)-1,2,5-oxadiazole 2-oxide (5): Compound **4** (2.2 mmol, 514 mg) and 2-chloro-4-nitroimidazole (2 mmol, 295 mg) were added to 10 mL MeOH, and the triethylamine (4 mmol, 405 mg) was slowly added. The resulting mixture was stirred overnight at room temperature until the substrate disappeared, and then, the MeOH was removed under reduced pressure. Column chromatography of the residue on silica gel eluting with PE/EA (3:1) gave compound **5** as a pale-yellow solid, yield 450 mg (75%). *T*_dec_: 151.1 °C; ^1^H NMR (400 MHz, DMSO) δ 8.74 (s, 1H), 8.49 (s, 1H), 3.23 (s, 3H), 3.12 (s, 3H). ^13^C NMR (100 MHz, DMSO) δ 160.0, 158.0, 157.7, 150.1, 143.3, 112.3, 103.3, 41.2, 35.3. IR (cm^−1^) 3184, 2925, 1719, 1636, 1610, 1539, 1516, 1434, 1392, 1331, 1294, 1274, 1213, 1127, 1110, 989, 896, 873, 849, 786, 732, 599, 587, 416. Elemental analysis (%) for C_9_H_8_N_8_O_5_ (308.21): calcd: C 35.07, H 2.62, N 36.36. Found: C 34.65, H 2.45, N 35.67. HRMS (ESI) m/z calcd for C_9_H_9_N_8_O_5_^+^ (M+H)^+^ 309.06904, found 309.06897 (Figures S2, S3 and S23).

4-Amino-3-(6-nitroimidazo [1,2-d][1,2,4]oxadiazol-3-yl)-1,2,5-oxadiazole 2-oxide (6): Compound **5** (308 mg, 1.0 mmol) was added to a mixture of concentrated HCl (2 mL) and water (6 mL), and the suspension was stirred at 25 °C for 48 h. The solid was collected by filtration and washed with ice water (10 mL) to give compound **6** as a yellow solid, yield 182 mg (72%). *T*_dec_: 163.6 °C; ^1^H NMR (400 MHz, DMSO) δ 8.41 (s, 1H), 6.86 (s, 2H). ^13^C NMR (100 MHz, DMSO) δ 157.9, 155.5, 150.4, 143.2, 111.6, 101.1. IR (cm^−1^) 3466, 3365, 3164, 2923, 1629, 1590, 1537, 1484, 1366, 1285, 1214, 1046, 978, 963, 857, 804, 789, 767, 745, 721, 708, 648. Elemental analysis (%) for C_6_H_3_N_7_O_5_ (253.13): calcd: C 28.47, H 1.19, N 38.73. Found: C 28.02, H 1.34, N 38.27. HRMS (ESI) m/z calcd for C_6_H_4_N_7_O_5_^+^ (M+H)^+^ 254.02684, found 254.02690 (Figures S4, S5 and S20).

3-(5-Amino-1,2,4-oxadiazol-3-yl)-4-nitro-1,2,5-oxadiazole 2-oxide (7a): Compound **6** (253 mg, 1.0 mmol) was dispersed in 30% H_2_O_2_ (3.6 g), and 98% H_2_SO_4_ (4.5 mL) was added dropwise at 0 °C. Then, Na_2_WO_4_ (330 mg, 1 mmol) was added in portions, and the reaction mixture was stirred overnight at room temperature. After the reaction was completed by TLC monitoring, the reaction mixture was quenched with 15 mL ice water and extracted with EA. The organic phase was combined and washed with water and then dried with Na_2_SO_4_. After the organic solvent was removed, the residue was purified by flash column chromatography on silica gel eluting with PE/EA (4:1) to give compound **7a** as a pale-yellow solid, yield 150 mg (70%). *T*_dec_: 176.8 °C; ^1^H NMR (400 MHz, DMSO) δ 8.48 (s, 1H). ^13^C NMR (100 MHz, DMSO) δ 173.1, 158.0, 155.6, 103.4. IR (cm^−1^) 3441, 3140, 1678, 1633, 1572, 1537, 15034, 1386, 1313, 1229, 1097, 1070, 1049, 991, 960, 9078, 847, 791, 760, 665, 464, 418. **Elemental analysis** (%) for C_4_H_2_N_6_O_5_ (214.10): calcd: C 22.44, H 0.94, N 39.25. Found: C 22.75, H 1.00, N 38.82 (Figures S6 and S7).

4-Nitro-3-(6-nitroimidazo [1,2-d][1,2,4]oxadiazol-3-yl)-1,2,5-oxadiazole 2-oxide (7): Compound **6** (253 mg, 1.0 mmol) was dispersed in 30% H_2_O_2_ (3 mL), and 98% concentrated sulfuric acid (3.5 mL) was added dropwise at 0 °C. Then, Na_2_WO_4_ (330 mg, 1 mmol) was added in batches, and the reaction mixture was slowly raised to room temperature and stirred for 4 h. After the reaction was completed, the reaction mixture was poured into 15 mL ice water and extracted with ethyl acetate. The organic phase was combined and washed with water and then dried with anhydrous sodium sulfate. After the organic solvent was removed, the solid residue was purified by flash column chromatography on silica gel eluting with petroleum ether/ethyl acetate (3:1) to give compound **7** as a pale-yellow solid, yield 232 mg (82%). *T*_dec_: 170.3 °C; ^1^H NMR (400 MHz, DMSO) δ 8.74 (s, 1H). ^13^C NMR (100 MHz, DMSO) δ 158.2, 157.7, 150.7, 140.6, 111.4, 100.1. ^14^N NMR (40 MHz, DMSO) δ -35.53. IR (cm^−1^) 3187, 2923, 2853, 1640, 1604, 1587, 1563, 1517, 1450, 1488, 1347, 1310, 1209, 1141, 1092, 1067, 987, 965, 890, 855, 841, 801, 761, 704, 666, 613, 494. Elemental analysis (%) for C_6_HN_7_O_7_ (283.12): calcd: C 25.45, H 0.36, N 34.63. Found: C 25.43, H 0.44, N 33.73. HRMS (ESI) m/z calcd for C_6_H_2_N_7_O_7_^+^ (M+H)^+^ 284.00102, found 284.00015 (Figures S8–S10 and S21).

4,4′-(Diazene-1,2-diyl)bis(3-(6-nitroimidazo [1,2-d][1,2,4]oxadiazol-3-yl)-1,2,5-oxadiazole 2-oxide) (8): To a solution of pure compound **6** (253 mg, 1.0 mmol) in acetic acid (2.5 mL), aqueous sodium hypochlorite solution (6–14% available chlorine, 1.2 g) was added dropwise and stirred at 25 °C for 5 min. The reaction solution was quenched with a small amount of water, resulting in solid precipitation. Subsequently, the obtained solid was filtered, washed with water, and dried to obtain a crude product. Then, the solid residue was purified by flash column chromatography on silica gel eluting with petroleum ether/ethyl acetate/AcOH (5:1:0.025) to give compound **8** as a yellow solid, yield 198 mg (79%). *T*_dec_: 158.8 °C; ^1^H NMR (400 MHz, CD_3_CN) δ 8.09 (s, 2H). ^13^C NMR (100 MHz, CD_3_CN) δ 161.5, 159.1, 152.2, 141.7, 109.9, 99.8. IR (cm^−1^) 3158, 2924, 1634, 1601, 1539, 1487, 1362, 1278, 1123, 1068, 1005, 968, 900, 884, 855, 792, 777, 755, 714, 644, 611, 552, 510, 450. HRMS (ESI) m/z calcd for C_12_H_3_N_14_O_10_^+^ (M+H)^+^ 503.01511, found 503.01480 (Figures S11, S12 and S22).

4-(Chloro(hydroxyimino)methyl)-3-cyano-1,2,5-oxadiazole 2-oxide (9)

Prepared according to the literature [6]. Yield 74%.

4-Cyano-3-(6-nitroimidazo [1,2-d][1,2,4]oxadiazol-3-yl)-1,2,5-oxadiazole 2-oxide (10): 4-(chloro(hydroxyimino)methyl)-3-cyano-1,2,5-oxadiazole 2-oxide (1.1 mmol, 188 mg) and 2-chloro-4-nitroimidazole (1 mmol, 148 mg) were added to 10 mL dichloromethane, and the triethylamine (2 mmol, 203 mg) diluted with 1.5 mL of dichloromethane was slowly added at 25 °C. The resulting mixture was stirred overnight at room temperature until the substrate disappeared, and then, the dichloromethane was removed under reduced pressure. Column chromatography of the residue on silica gel eluting with PE/EA (5:1) gave the compound **10** as a white solid, yield 184 mg (70%). *T*_dec_: 181.8 °C; ^1^H NMR (400 MHz, DMSO) δ 9.01 (s, 1H). ^13^C NMR (100 MHz, DMSO) δ 158.3, 151.8, 142.9, 142.4, 110.2, 105.7, 96.7. IR (cm^−1^) 3137, 2254, 1630, 1612, 1594, 1538, 1493, 1468, 1429, 1366, 1283, 1240, 1105, 1042, 991, 962, 885, 855, 830, 792, 753, 722, 693, 574, 502, 470, 427. HRMS (ESI) m/z calcd for C_7_N_7_O_5_^−^ (M-H)^−^ 261.99664, found 261.99609 (Figures S13, S14 and S24).

4-(5-Amino-1,2,4-oxadiazol-3-yl)-3-(1H-tetrazol-5-yl)-1,2,5-oxadiazole 2-oxide (11): Compound **10** (263 mg, 1.0 mmol) was dissolved in water (10 mL), and sodium azide (130 mg, 2.0 mmol) and zinc chloride (204 mg, 1.5 mmol) were added. The solution was heated to 50 °C and stirred for overnight. The reaction mixture was cooled to ambient temperature and acidified with 2 N HCl to pH 1~2. The mixtures were extracted with ethyl acetate. After the combined organic phase was evaporated, the obtained solid was recrystallized to afford compound **11**, yield 159 mg (52%). *T*_dec_: 251.0 °C; ^1^H NMR (400 MHz, DMSO) δ 13.73 (s, 1H), 8.41 (s, 1H). ^13^C NMR (100 MHz, DMSO) δ 173.2, 170.9, 158.9, 147.2, 145.5, 106.6. IR (cm^−1^) 3412, 3288, 3211, 3057, 3012, 1785, 1677, 1629, 1522, 1371, 1287, 1220, 1109, 1056, 993, 830, 541. HRMS (ESI) m/z calcd for C_5_H_2_N_9_O_3_^−^ (M-H)^−^ 236.02806, found 236.02824 (Figures S15 and S16).

4-(((Dimethylamino)methylene)amino)-3-(6-nitro-[1,2,4]triazolo [1,5-d][1,2,4]oxadiazol-3-yl)-1,2,5-oxadiazole 2-oxide (5a): Compound **4** (2.2 mmol, 514 mg) and 5-bromo-3-nitro-1*H*-1,2,4-triazole (2 mmol, 386 mg) were added to 10 mL dichloromethane, and the triethylamine (4 mmol, 405 mg) was slowly added at 25 °C. The resulting mixture was stirred overnight at room temperature until the substrate disappeared, and then, the solvent was removed under reduced pressure. Column chromatography of the residue on silica gel eluting with PE/EA (4:1) gave the compound **5a** as a pale-yellow solid, yield 278 mg (45%). *T*_dec_: 116.4 °C; ^1^H NMR (400 MHz, CD_3_CN) δ 8.34 (s, 1H), 3.14 (s, 3H), 3.11 (s, 3H). ^13^C NMR (100 MHz, CD_3_CN) δ 167.8, 167.3, 160.7, 157.3, 143.6, 102.2, 41.31, 35.1. IR (cm^−1^) 2930, 1637, 1607, 1587, 1552, 1525, 1434, 1399, 1298, 1268, 1112, 1062, 964, 870, 848, 837, 753, 737, 633, 591, 473. HRMS (ESI) m/z calcd for C_8_H_8_N_9_O_5_^+^ (M+H)^+^ 310.06429, found 310.06470 (Figures S17, S18 and S25).

4,4′-(1,4,2,5-Dioxadiazine-3,6-diyl)bis(3-cyano-1,2,5-oxadiazole 2-oxide) (12): 3-(chloro(hydroxyimino)methyl)-4-cyano-1,2,5-oxadiazole 2-oxide (1.1 mmol, 188 mg) were added to 10 mL dichloromethane, and the triethylamine (2 mmol, 203 mg) diluted with 1.5 mL of dichloromethane was slowly added at 25 °C. The resulting mixture was stirred overnight at room temperature until the substrate disappeared, and then, the solvent was removed under reduced pressure. Column chromatography of the residue on silica gel eluting with PE/EA (10:1) gave compound **12** as a pale-yellow solid, yield 94 mg (62%). *T*_dec_: 170.4 °C; ^13^C NMR (100 MHz, DMSO) δ 152.1, 145.1, 105.9, 97.2. IR (cm^−1^) 2923, 2852, 2258, 1747, 1626, 1522, 1483, 1347, 1228, 1046, 1025, 1006, 964, 896, 838, 739, 633, 577, 538, 507, 439, 406. HRMS (ESI) m/z calcd for C_8_HN_8_O_6_^+^ (M+H)^+^ 305.00190, found 305.00146 (Figures S19 and S26).

## 4. Conclusions

In summary, three newly designed energetic compounds based on the combination of furoxan and oxa-[5,5]bicyclic ring were synthesized and deeply characterized. Compound **7** possesses a high density of 1.810 g cm^−3^ and is thermally stable up to 170.3 °C. Because of the introduction of nitrofuroxan, the synthesized nitro compound **7** exhibits good oxygen balances and detonation properties (OB, 2.83%; *Dv*, 8565 m s^−1^; *P*, 31.9 GPa) comparable to that of RDX. These novel structures represent the first examples of a combination of the furoxan and oxa-[5,5]bicyclic structures, which would open a new avenue for the construction of new high-energy-density material.

## Figures and Tables

**Table 1:** Physicochemical and energetic properties of compounds **6**, **7**, and **8**.

Compound	*T_d_ ^a^* (°C)	*d ^b^* (g cm^−3^)	Δ*H_f_ ^c^* (kJ mol^−1^)	*D ^d^* (m s^−1^)	*P ^e^* (Gpa)	Q *^f^* (kJ kg^−1^)	Detonation Temperature *^g^* (K)	IS *^h^* (J)	FS *^i^* (N)	OB *^j^* (%)	[N+O] *^k^* (%)
6	163.6	1.807	370.7	7960	26.1	4871	3597	5	80	−15.8	70.3
7	170.3	1.810	454.9	8565	31.9	5812	4490	3	84	2.83	74.2
8	158.8	1.789	1212.7	8241	28.6	5574	4292	3	84	−9.56	70.9
RDX	204	1.806	70.3	8795	34.9	5742	3739	7.5	120	0	81.1

*^a^* Decomposition temperature from DSC (5 °C min^−1^). *^b^* Crystal density (298K). *^c^* Calculated molar enthalpy of formation in solid state. *^d^* Detonation velocity calculated with EXPLO5 V6.05. *^e^* Detonation pressure calculated with EXPLO5 V6.05. *^f^* Heat of detonation calculated with EXPLO5 V6.05. *^g^* Detonation temperature calculated with EXPLO5 V6.05. *^h^* Impact sensitivity. *^i^* Friction sensitivity. *^j^* Oxygen balances based on CO as the product for C_a_H_b_O_c_N_d_, Ω_CO_ (%) = 1600 (c–a–b/2)/Mw; Mw = molecular weight. *^k^* Nitrogen–oxygen content.

## Data Availability

Not applicable.

## Supplementary Materials

The following supporting information can be downloaded at: https://www.mdpi.com/article/10.3390/ijms24108846/s1.
