# Supplementary material for: Enhanced Energetic Performance via the Combination of Furoxan and Oxa-[5,5]bicyclic Structures
Source: Int J Mol Sci. 2023 May 16;24(10):8846. doi: 10.3390/ijms24108846 (PMC10218447; doi:10.3390/ijms24108846)
Supplement: Supplementary file 1 [file ijms-24-08846-s001.zip › ijms-2388371-supplementary.pdf]

Supporting Information for

Enhanced Energetic Performance via the Combination of Furoxan and Oxa-[5,5]bicyclic Structures

Qi Zhang[a,b], Xun Zhang[a,b], Siping Pang*[a] and Chunlin He*[a,b,c]

[a] School of Materials Science & Engineering, Beijing Institute of Technology, Beijing 100081, China;

[b] Experimental Center of Advanced Materials, Beijing Institute of Technology, Beijing 100081, China;

[c] Yangtze Delta Region Academy of Beijing Institute of Technology, Jiaxing 314019, China;

E-mail: pangsp@bit.edu.cn, chunlinhe@bit.edu.cn.

Contents

I. X-Ray crystallographic data..S2

II. DFT calculations..S13

III. Copies of ^{1}H-NMR and ^{13}C-NMR spectra..S15

IV. DSC curves..S24

I. X-Ray crystallographic data

Table S1. Crystal Structure of 4-(((dimethylamino)methylene)amino)-3-(6-nitroimidazo[1,2-d][1,2,4]oxadiazol-3-yl)-1,2,5-oxadiazole 2-oxide (**5**)

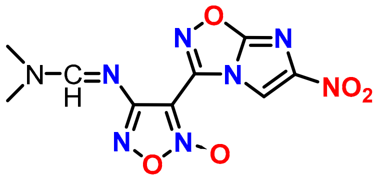

(CCDC No. **2237350**)

Empirical formula	$C_9H_8N_8O_5$
Temperature	170.0 K
Wavelength	0.71073 Å
Unit cell dimensions	a = 6.6056(3) Å<br>b = 9.7316(3) Å<br>c = 19.4758(8) Å<br>alpha = 90 deg.<br>beta = 90 deg.<br>gamma = 90 deg.
Volume	1251.96 (9) $Å^3$
Z	4
Calculated density	1.635 g/cm^3
Absorption coefficient	0.137 mm^{-1}
F(000)	632.0
Crystal size	0.11 x 0.05 x 0.03 mm
2Θ range for data collection	4.68 to 52.864 deg.
Reflections collected / unique	9294 / 2562[R(int) = 0.0395]
Data / restraints / parameters	2562 / 0 / 202
Goodness-of-fit on F^2	1.066
Final R indexes [I>2sigma(I)]	R1 = 0.0341, wR2 = 0.0755
Final R indexes (all data)	R1 = 0.0439, wR2 = 0.0818

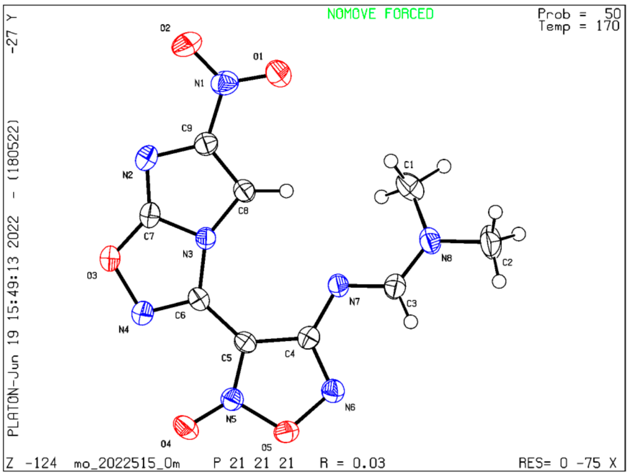

Growth in MeOH at room temperature

Table S2. Crystal Structure of 4-amino-3-(6-nitroimidazo[1,2-d][1,2,4]oxadiazol-3-yl)-1,2,5-oxadiazole 2-oxide (**6**)

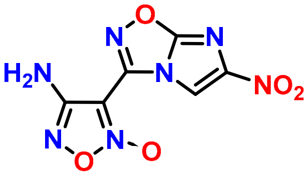

(CCDC No. **2237351**)

Empirical formula	2 ($C_6H_3N_7O_5$)
Temperature	298.0 K
Wavelength	0.71073 Å
Unit cell dimensions	a = 8.531 (2) Å<br>b = 22.849 (6) Å<br>c = 9.967 (3) Å<br>alpha = 90 deg.<br>beta = 106.721 (9) deg.<br>gamma = 90 deg.
Volume	1860.6 (9) Å^3
Z	4
Calculated density	1.807 g/cm^3
Absorption coefficient	0.159 mm^{-1}
F(000)	1024.0

Crystal size	0.07 x 0.03 x 0.02 mm
2Θ range for data collection	4.624 to 52.842 deg.
Reflections collected / unique	10910 / 3785 [R(int) = 0.1248]
Data / restraints / parameters	3785 / 0 / 326
Goodness-of-fit on F^2	1.015
Final R indexes [I>2sigma(I)]	R1 = 0.0769, wR2 = 0.1718
Final R indexes (all data)	R1 = 0.1604, wR2 = 0.2268

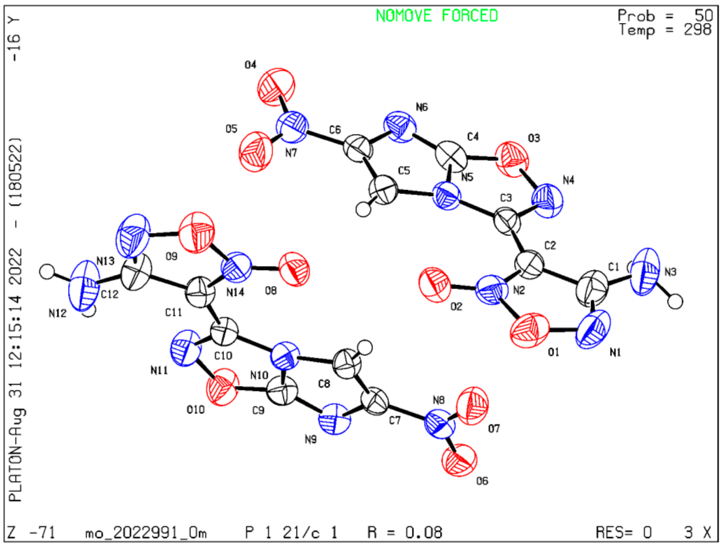

Growth in EA/PE at room temperature

Table S3. Crystal Structure of 4-nitro-3-(6-nitroimidazo[1,2-d][1,2,4]oxadiazol-3-yl)-1,2,5-oxadiazole 2-oxide (**7**)

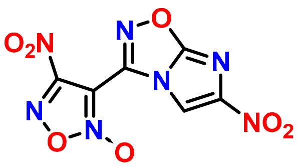

(CCDC No. **2237352**)

Empirical formula	$C_6HN_7O_7$
Temperature	298.0 K
Wavelength	0.71073 Å
Unit cell dimensions	a = 11.993 (6) Å<br>b = 13.077 (11) Å<br>c = 13.250 (6) Å<br>alpha = 90 deg.<br>beta = 90 deg.<br>gamma = 90 deg.
Volume	2078 (2) $Å^3$
Z	8
Calculated density	1.810 g/cm^3
Absorption coefficient	0.167 mm^{-1}
F(000)	1136.0
Crystal size	0.12 x 0.08 x 0.05 mm
2Θ range for data collection	5.54 to 52.916 deg.
Reflections collected / unique	7959 / 2093 [R(int) = 0.0851]
Data / restraints / parameters	2093 / 443 / 262
Goodness-of-fit on F^2	0.980
Final R indexes [I>2sigma(I)]	R1 = 0.0849, wR2 = 0.2197
Final R indexes (all data)	R1 = 0.1828, wR2 = 0.2795

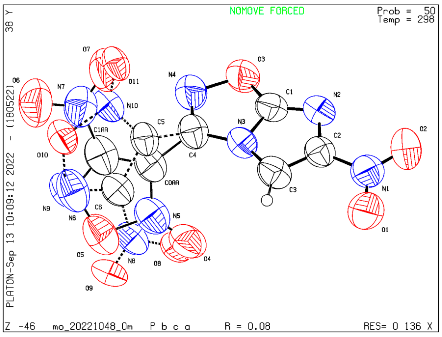

Growth in EA/PE at room temperature

Table S4. Crystal Structure of 3-(5-amino-1,2,4-oxadiazol-3-yl)-4-nitro-1,2,5-oxadiazole 2-oxide (**7a**)

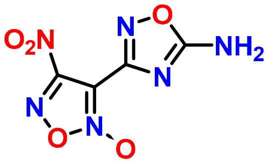

(CCDC No. **2253262**)

Empirical formula	$C_4H_2N_6O_5$
Temperature	298.0 K
Wavelength	0.71073 Å
Unit cell dimensions	a = 6.1384 (8) Å<br>b = 8.3578 (13) Å<br>c = 15.223 (2) Å<br>alpha = 90 deg.<br>beta = 97.454 (4) Å<br>gamma = 90 deg.
Volume	774.4 (2) $Å^3$
Z	4
Calculated density	1.837 g/cm^3
Absorption coefficient	0.169 mm^{-1}
F(000)	432.0
Crystal size	0.15 x 0.06 x 0.04 mm

2Θ range for data collection	5.398 to 54.26 deg.
Reflections collected / unique	8835 / 1706 [R(int) = 0.0982]
Data / restraints / parameters	1706 / 0 / 136
Goodness-of-fit on F^2	0.981
Final R indexes [I>2sigma(I)]	R1 = 0.0522, wR2 = 0.1188
Final R indexes (all data)	R1 = 0.1031, wR2 = 0.157
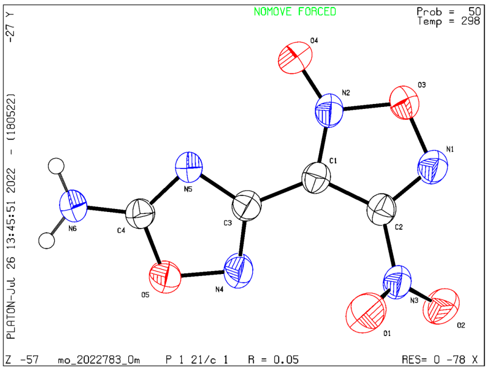	
Growth in EA/PE at room temperature	

Table S5. Crystal Structure of 3-cyano-4-(6-nitroimidazo[1,2-d][1,2,4]oxadiazol-3-yl)-1,2,5-oxadiazole 2-oxide (**10**)

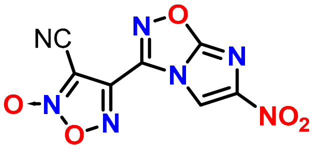

(CCDC No. **2252471**)

Empirical formula	$C_7HN_7O_5$
Temperature	300.0 K
Wavelength	0.71073 Å
Unit cell dimensions	a = 13.430 (7) Å<br>b = 5.669 (3) Å<br>c = 13.214 (6) Å<br>alpha = 90 deg.<br>beta = 90 deg.<br>gamma = 90 deg.
Volume	1006 (9) $Å^3$

Z	4
Calculated density	1.737 g/cm^3
Absorption coefficient	0.151 mm^{-1}
F(000)	528.0
Crystal size	0.08 x 0.03 x 0.01 mm
2Θ range for data collection	6.066 to 52.88 deg.
Reflections collected / unique	4196 / 1824 [R(int) = 0.0953]
Data / restraints / parameters	1824 / 8 / 173
Goodness-of-fit on F^2	1.026
Final R indexes [I>2sigma(I)]	R1 = 0.0668, wR2 = 0.1302
Final R indexes (all data)	R1 = 0.1855, wR2 = 0.1822
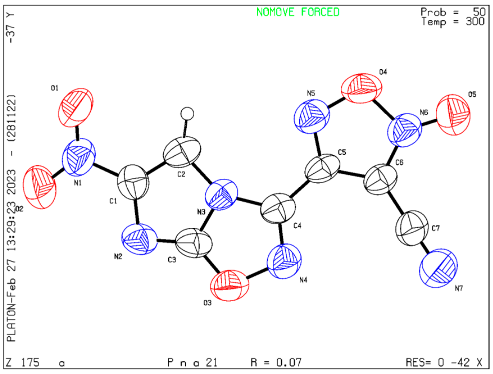	
Growth in EA/PE at room temperature	

Table S6. Crystal Structure of 4-(5-amino-1,2,4-oxadiazol-3-yl)-3-(1H-tetrazol-5-yl)-1,2,5-oxadiazole 2-oxide (**11**)

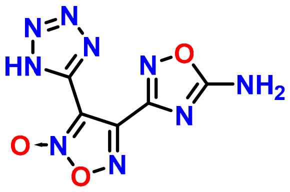

(CCDC No. **2252472**)

Empirical formula	$C_5H_3N_9O_3$
Temperature	298.0 K

Wavelength	0.71073 Å
Unit cell dimensions	a = 8.1992 (15) Å<br>b = 15.010 (3) Å<br>c = 7.2284 (10) Å<br>alpha = 90 deg.<br>beta = 101.900 (5) Å<br>gamma = 90 deg.
Volume	870.5 (3) $Å^3$
Z	4
Calculated density	1.810 g/cm^3
Absorption coefficient	0.153 mm^{-1}
F(000)	480.0
Crystal size	0.13 x 0.06 x 0.03 mm
2Θ range for data collection	5.078 to 52.728 deg.
Reflections collected / unique	4576 / 1718 [R(int) = 0.0436]
Data / restraints / parameters	1718 / 0 / 166
Goodness-of-fit on F^2	1.070
Final R indexes [I>2sigma(I)]	R1 = 0.0443, wR2 = 0.0874
Final R indexes (all data)	R1 = 0.0777, wR2 = 0.1045
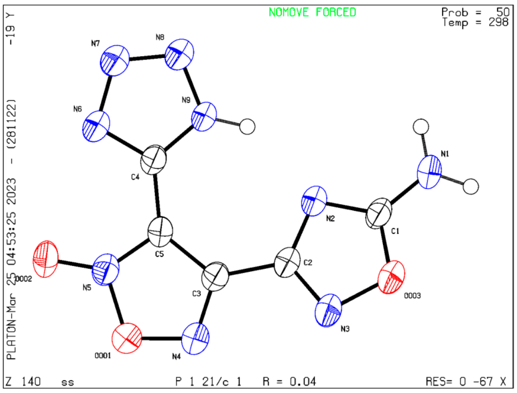	
Growth in EA/PE at room temperature	

Table S7. Crystal Structure of 4-(((dimethylamino)methylene)amino)-3-(6-nitro-[1,2,4]triazolo[1,5-d][1,2,4]oxadiazol-3-yl)-1,2,5-oxadiazole 2-oxide (**5a**)

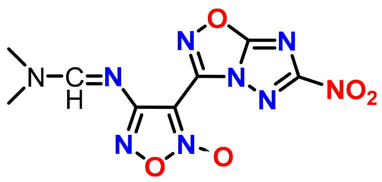

(CCDC No. 2245807)

Empirical formula	$C_8H_7N_9O_5$
Temperature	170.0 K
Wavelength	0.71073 Å
Unit cell dimensions	a = 4.2736 (10) Å<br>b = 15.607 (4) Å<br>c = 18.157 (4) Å<br>alpha = 90 deg.<br>beta = 92.863 (6) Å<br>gamma = 90 deg.
Volume	1209.6 (5) $Å^3$
Z	4
Calculated density	1.698 g/cm^3
Absorption coefficient	0.144 mm^{-1}
F(000)	632.0
Crystal size	0.11 x 0.04 x 0.02 mm
2Θ range for data collection	4.492 to 53.026 deg.
Reflections collected / unique	8642 / 2500 [R(int) = 0.0875]
Data / restraints / parameters	2500 / 0 / 202
Goodness-of-fit on F^2	1.041
Final R indexes [I>2sigma(I)]	R1 = 0.0948, wR2 = 0.2442
Final R indexes (all data)	R1 = 0.1814, wR2 = 0.2948

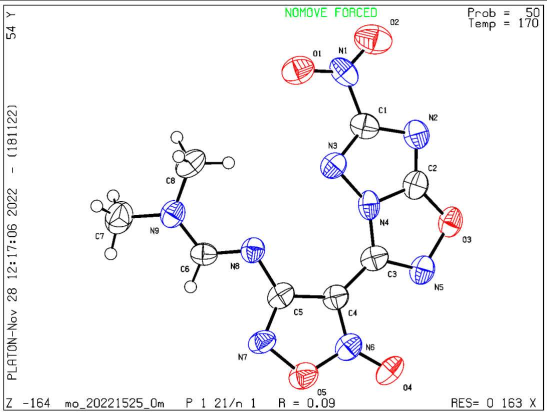
Growth in EA/PE at room temperature

Table S8. Crystal Structure of 4-amino-3-carbamoyl-1,2,5-oxadiazole 2-oxide (**6b**)

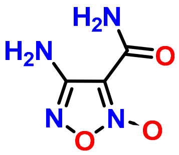

(CCDC No. **2245809**)

Empirical formula	$C_3H_4N_4O_3$
Temperature	298.0 K
Wavelength	0.71073 Å
Unit cell dimensions	a = 4.8612 (10) Å<br>b = 4.9098 (10) Å<br>c = 23.154 (5) Å<br>alpha = 90 deg.<br>beta = 93.448 (7) Å<br>gamma = 90 deg.
Volume	551.6 (2) $Å^3$
Z	4
Calculated density	1.735 g/cm^3
Absorption coefficient	0.154 mm^{-1}
F(000)	296.0
Crystal size	0.15 x 0.08 x 0.05 mm
2Θ range for data collection	7.052 to 52.858 deg.

Reflections collected / unique	1134 / 1134 [R(sigma) = 0.0752]
Data / restraints / parameters	1134 / 0 / 92
Goodness-of-fit on F^2	1.050
Final R indexes [I>2sigma(I)]	R1 = 0.0787, wR2 = 0.1746
Final R indexes (all data)	R1 = 0.1374, wR2 = 0.2072
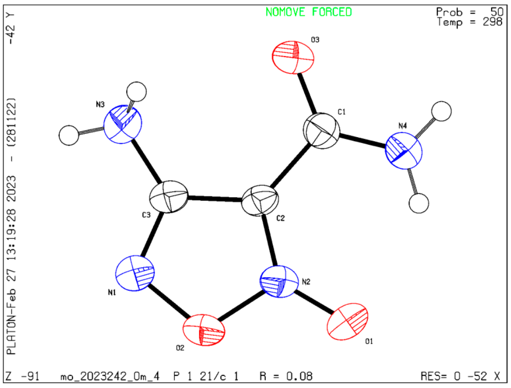	
Growth in EA/PE at room temperature	

Table S9. Crystal Structure of 4,4'-(1,4,2,5-dioxadiazine-3,6-diyl)bis(3-cyano-1,2,5-oxadiazole 2-oxide) (**12**)

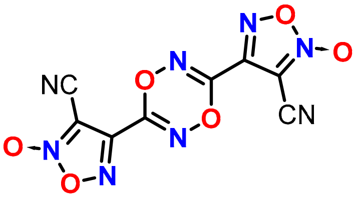

(CCDC No. **2252474**)

Empirical formula	$C_8N_8O_6$
Temperature	170.0 K
Wavelength	1.54178 Å
Unit cell dimensions	a = 9.3958 (9) Å<br>b = 6.1391 (6) Å<br>c = 20.068 (3) Å<br>alpha = 90 deg.<br>beta = 90 deg<br>gamma = 90 deg.

Volume	1157.6 (2) Å^3
Z	4
Calculated density	1.745 g/cm^3
Absorption coefficient	1.357 mm^{-1}
F(000)	608.0
Crystal size	0.11 x 0.04 x 0.02 mm
2Θ range for data collection	8.812 to 125.012 deg.
Reflections collected / unique	3616 / 1377 [R(int) = 0.0587]
Data / restraints / parameters	1377 / 1 / 200
Goodness-of-fit on F^2	1.083
Final R indexes [I>2sigma(I)]	R1 = 0.0561, wR2 = 0.1271
Final R indexes (all data)	R1 = 0.0744, wR2 = 0.1338
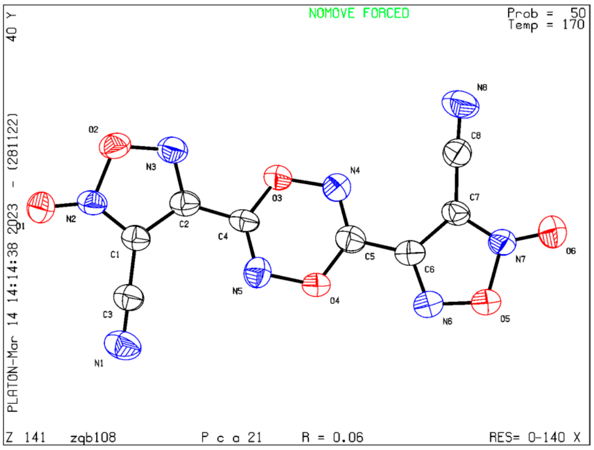	
Growth in EA/PE at room temperature	

II. DFT calculations

All quantum chemical calculations were carried out using the Gaussian 09 program package and visualized by GaussView 5.0. The geometric optimization and frequency analyses of the structures were carried out using the B3LYP functional with 6-311+g(d,p) basis set, and single energy points were calculated at the M062X/6-311g(d,p) level. All of the optimized structures were characterized to be true local energy minima on the potential energy surface without imaginary frequencies. The heat of formation was determined using an isodesmic reaction (Fig. S1). The heat of sublimation (ΔH_{sub}) was calculated according to Trouton's rule according to eqn (1), where T represents either the melting point or the decomposition temperature when no melting was observed.

$$\Delta H_{\mathrm{sub}} = 0.188/\mathrm{kJ\ mol^{-1}K^{-1}} \times T \qquad (1)$$

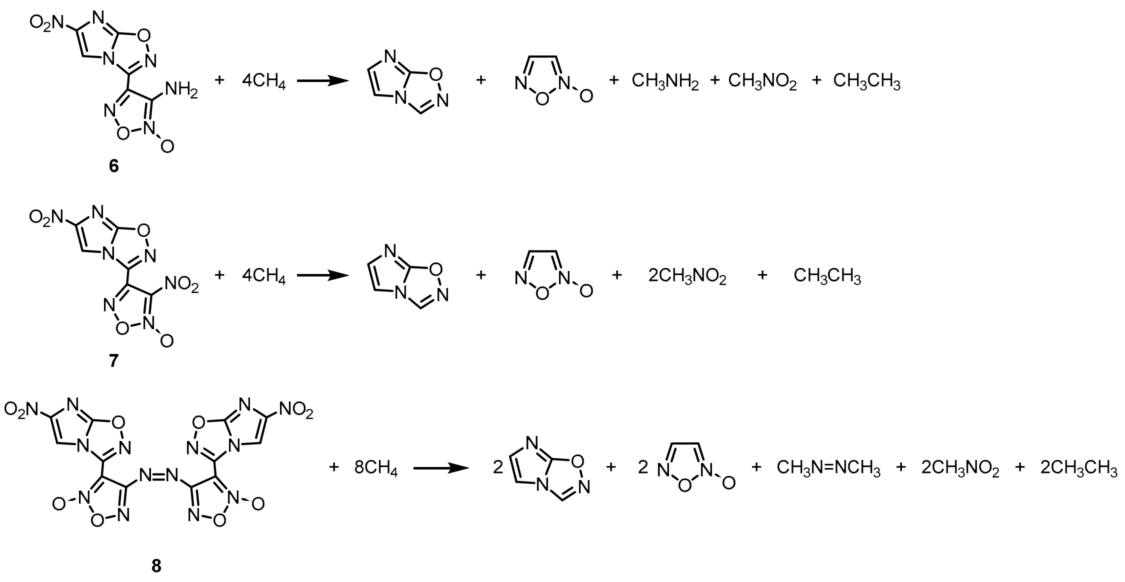

Figure S1. Isodesmic reaction for calculating heat of formation for compound **6-8**.

Table S10. Calculated (B3LYP/6-311+g(d,p)//M062X/6-311g(d,p)) total energy(E_0), zero-point energy (ZPE), values of the correction (Hr), and enthalpy of formation in gas-state (HOF) for **6-8**.

Compound	ZPE	Hr	E_0	Corrected E_0	$\Delta_f H_{gas}$ (kJ mol^{-1})	ΔH_{sub}	$\Delta_f H_{solid}$ (kJ mol^{-1})
**6**	0.123641	0.138521	-989.3795238	-989.2459484	452.8502013	82.1	370.7
**7**	0.108749	0.124931	-1138.464143	-1138.343562	538.3179269	83.4	454.9
**8**	0.202484	0.230978	-1976.258304	-1976.035426	1291.733024	79.0	1212.7
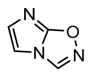	0.074939	0.081037	-393.573137	-393.4950976	264.9108188		
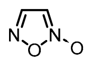	0.049235	0.054568	-337.1509835	-337.0983849	198.492395		
CH_3NH_2	0.063795	0.068178	-95.8302854	-95.7646592	-22.93089172		
CH_3NO_2	0.049659	0.054934	-244.9716383	-244.9186907	-85.58792832		
CH_4	0.044532	0.048345	-40.4966314	-40.45006768	-77.71645557		
CH_3CH_3	0.074312	0.078744	-79.7968535	-79.72108198	-86.07561695		
$CH_3N=NCH_3$	0.084056	0.090166	-189.2312905	-189.1444867	152.0814537		

III. Figures of ^{1}H-NMR, ^{13}C-NMR spectra

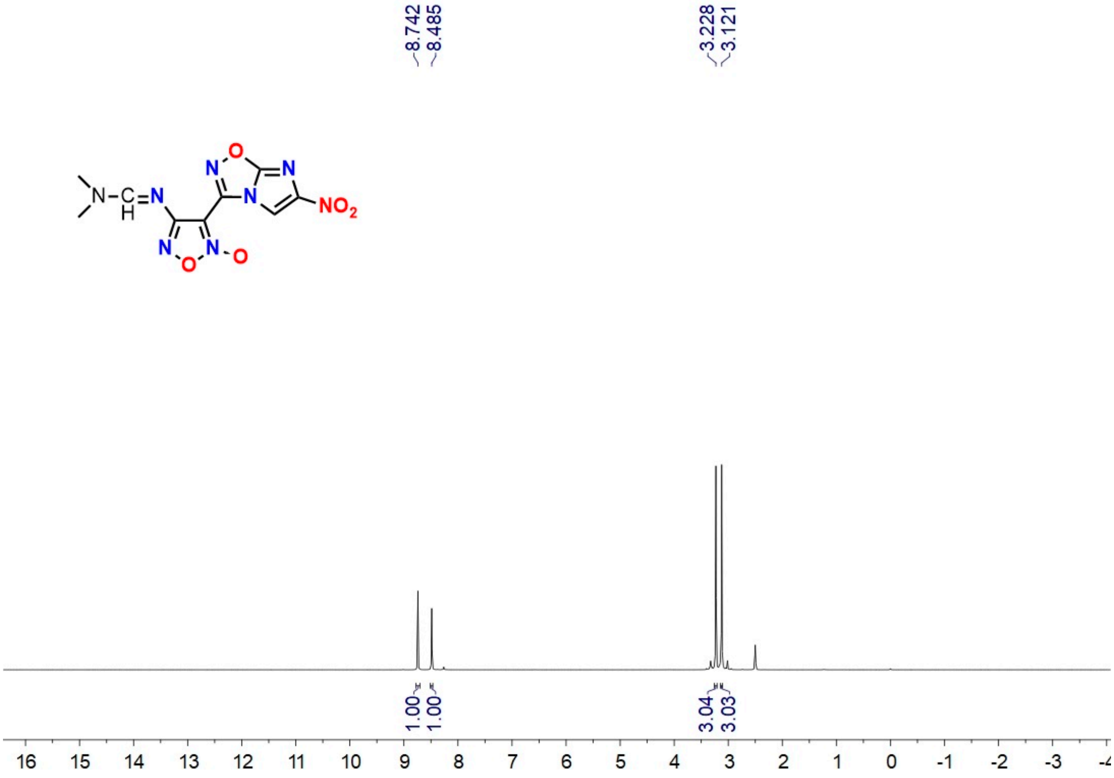

Figure S2. ^{1}H NMR Spectrum of **5** (DMSO, 400 MHz)

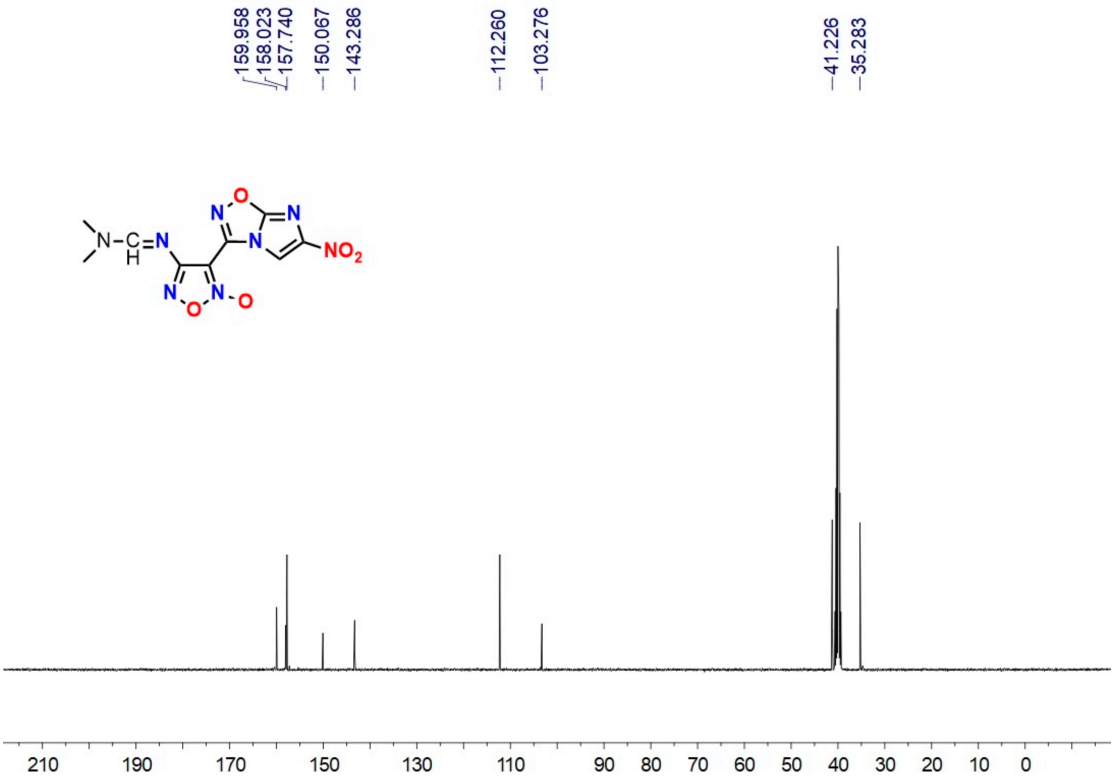

Figure S3. ^{13}C NMR Spectrum of **5** (DMSO, 100 MHz)

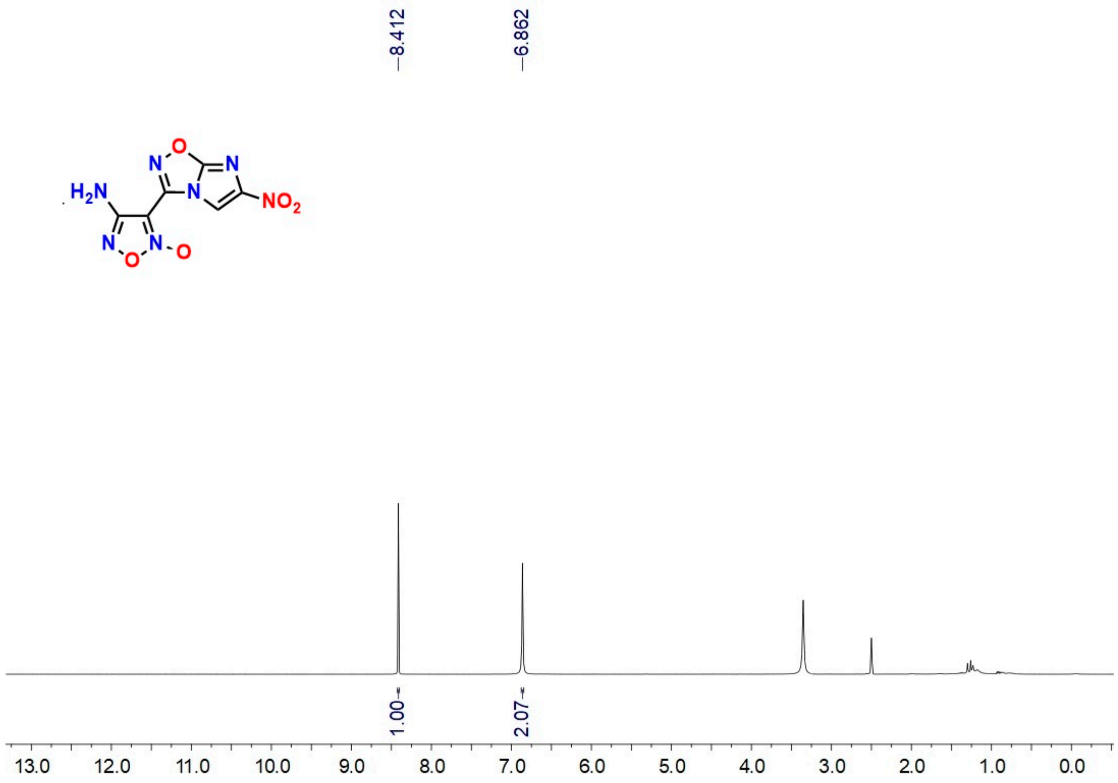

Figure S4. ^{1}H NMR Spectrum of **6** (DMSO, 400 MHz)

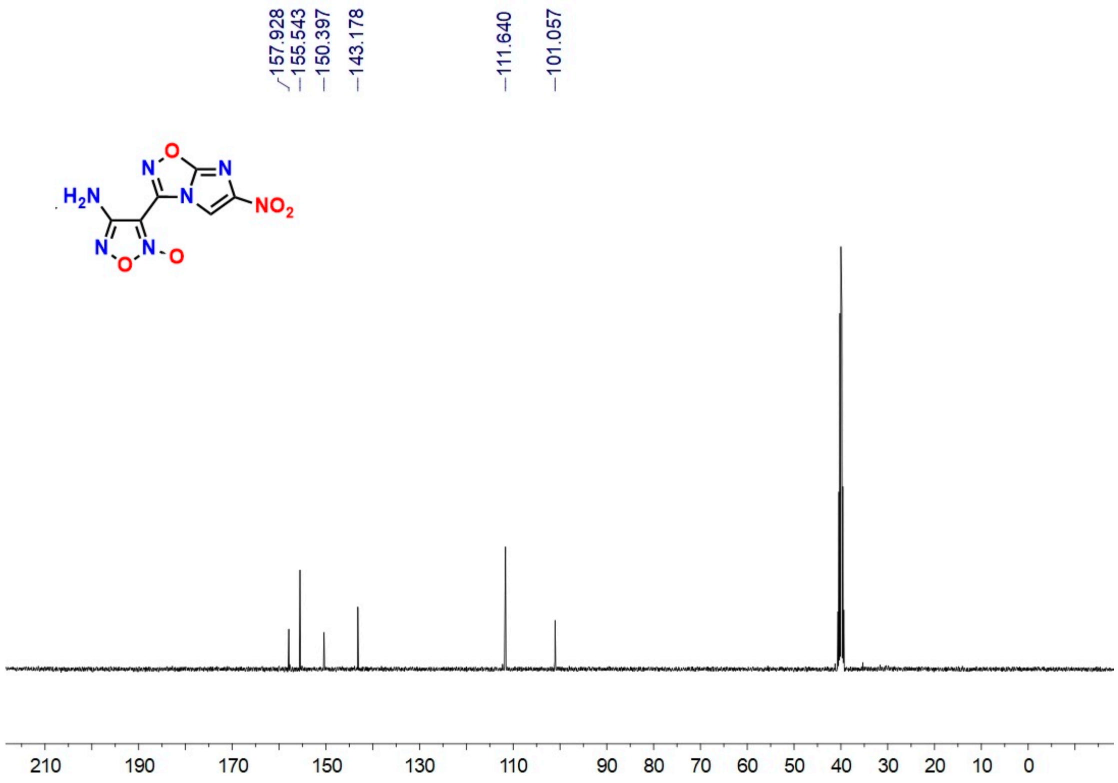

Figure S5. ^{13}C NMR Spectrum of **6** (DMSO, 100 MHz)

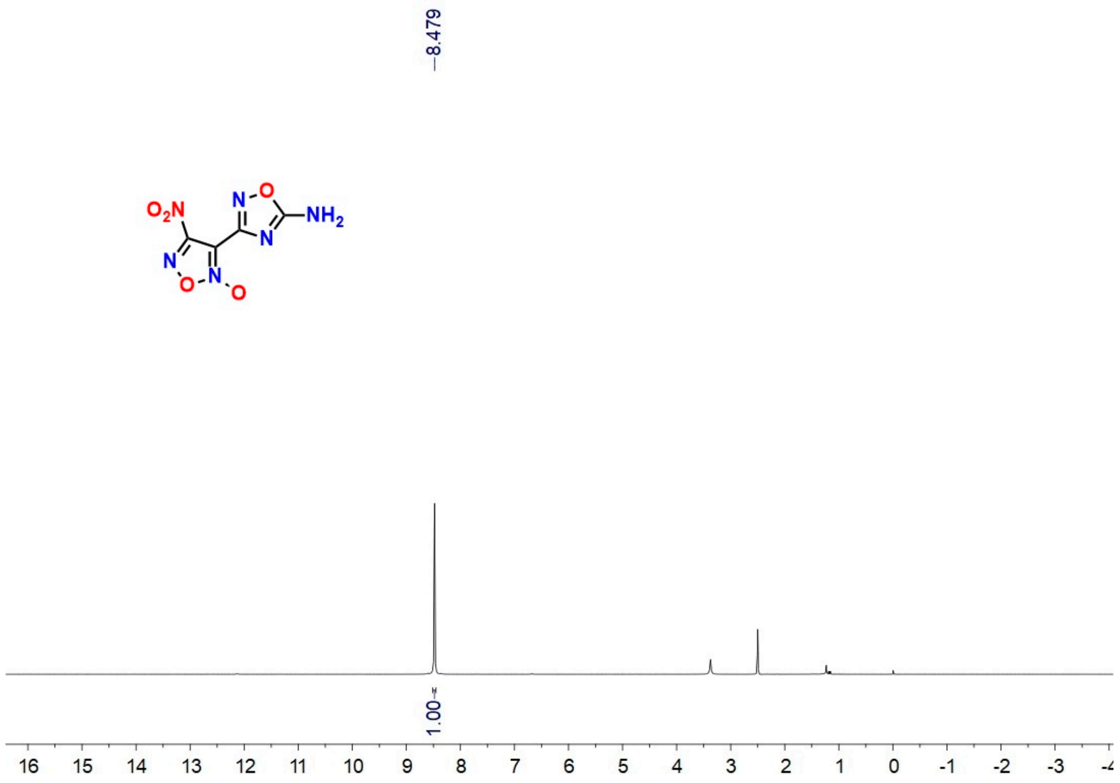

Figure S6. ^1H NMR Spectrum of **7a** (DMSO, 400 MHz)

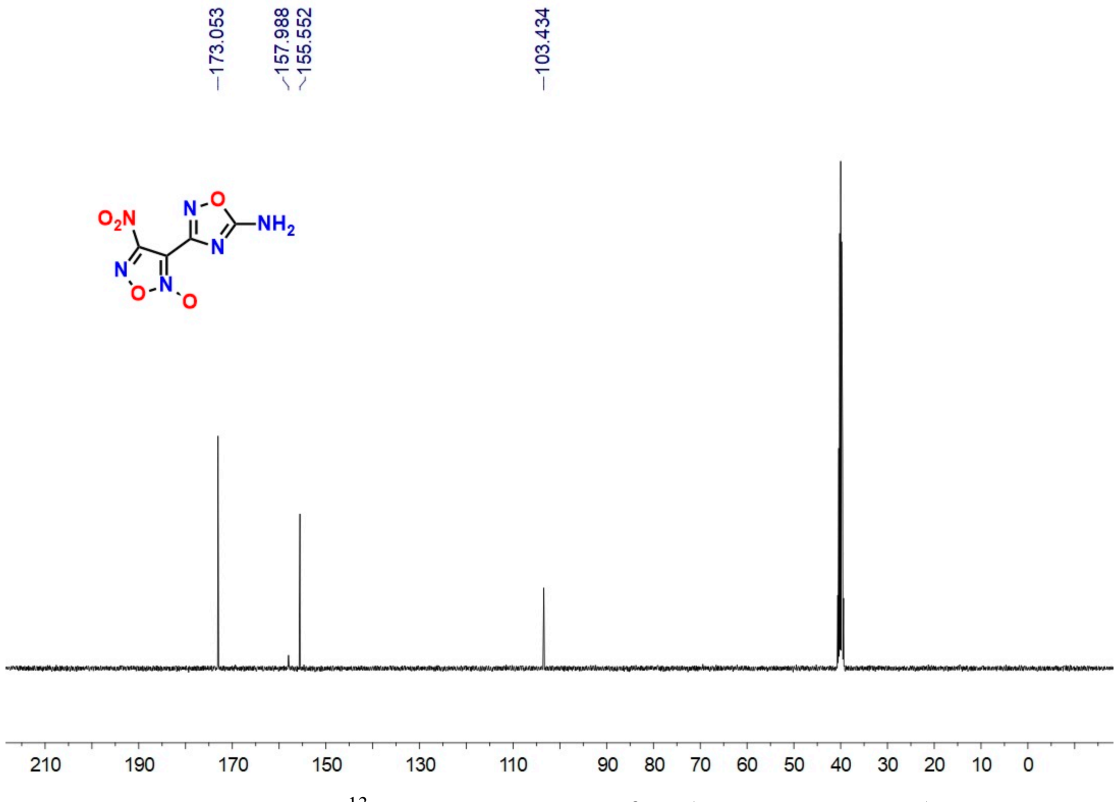

Figure S7. ^{13}C NMR Spectrum of **7a** (DMSO, 100 MHz)

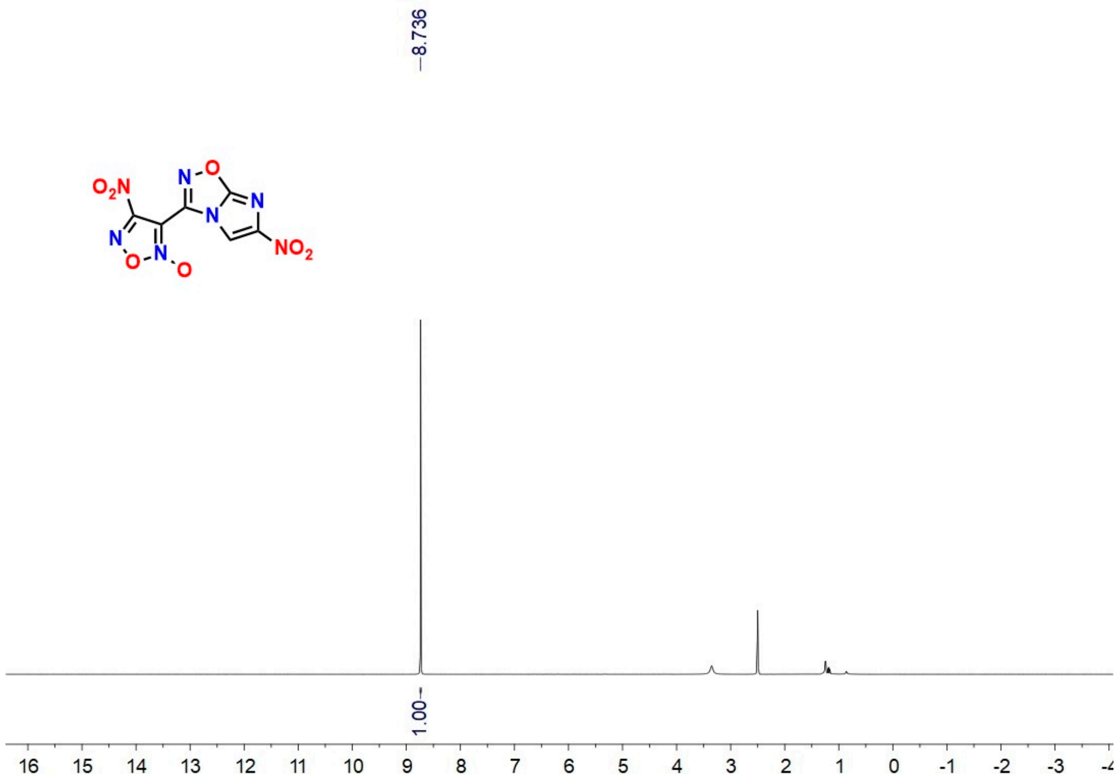

Figure S8. ^{1}H NMR Spectrum of **7** (DMSO, 400 MHz)

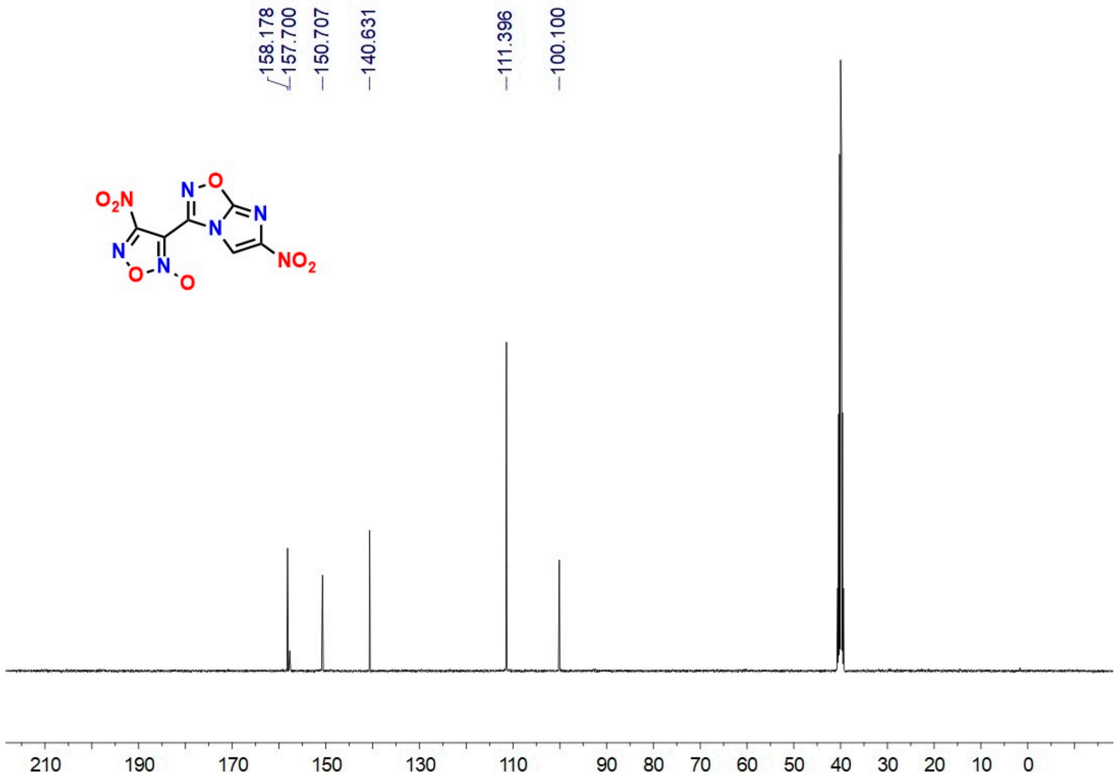

Figure S9. ^{13}C NMR Spectrum of **7** (DMSO, 100 MHz)

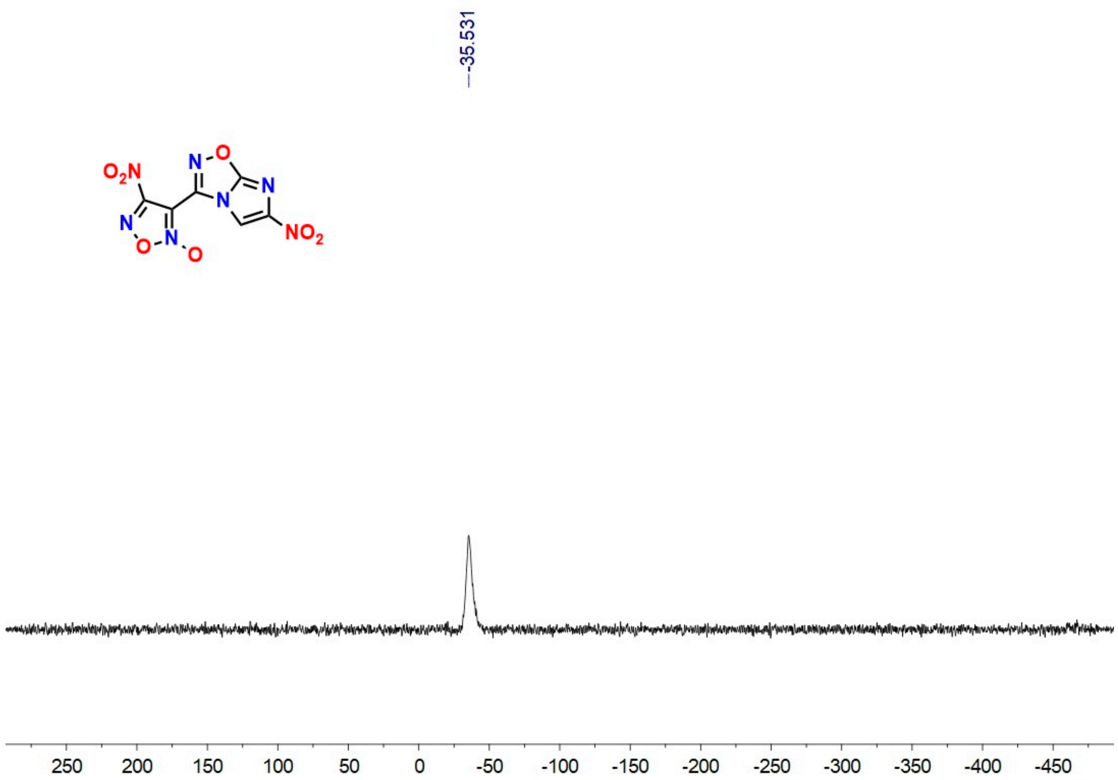

Figure S10. ^{14}N NMR Spectrum of **7** (DMSO, 40 MHz)

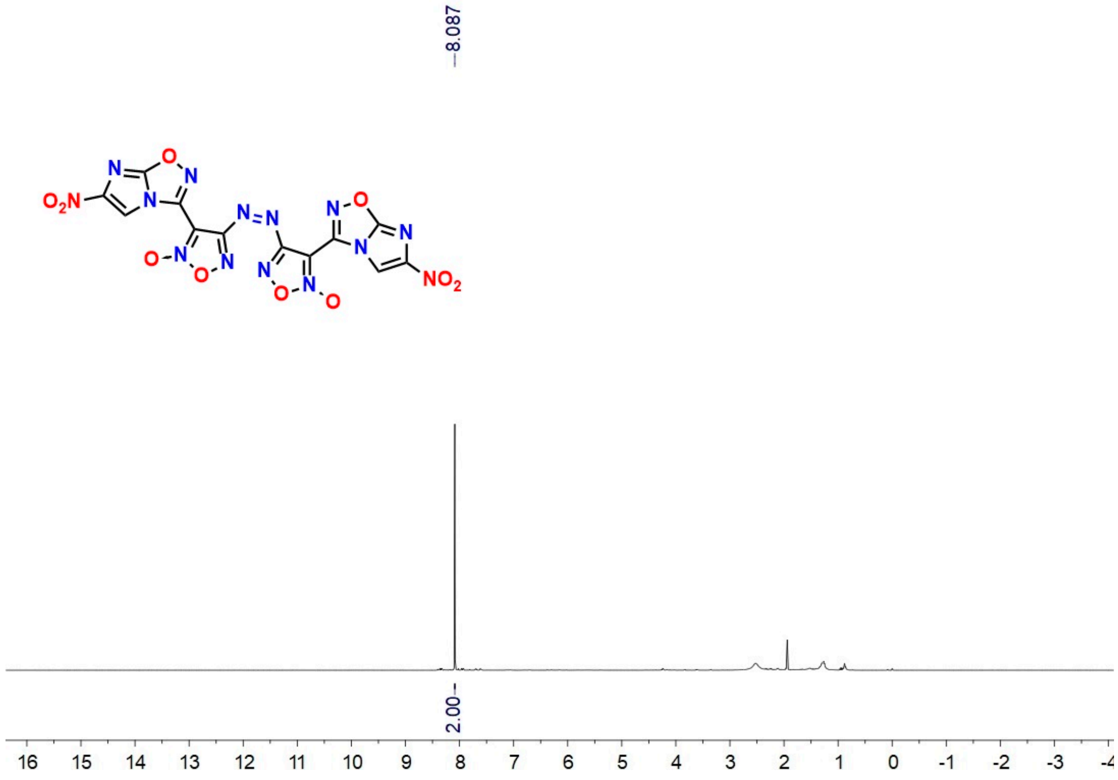

Figure S11. ^{1}H NMR Spectrum of **8** (CD_3CN, 400 MHz)

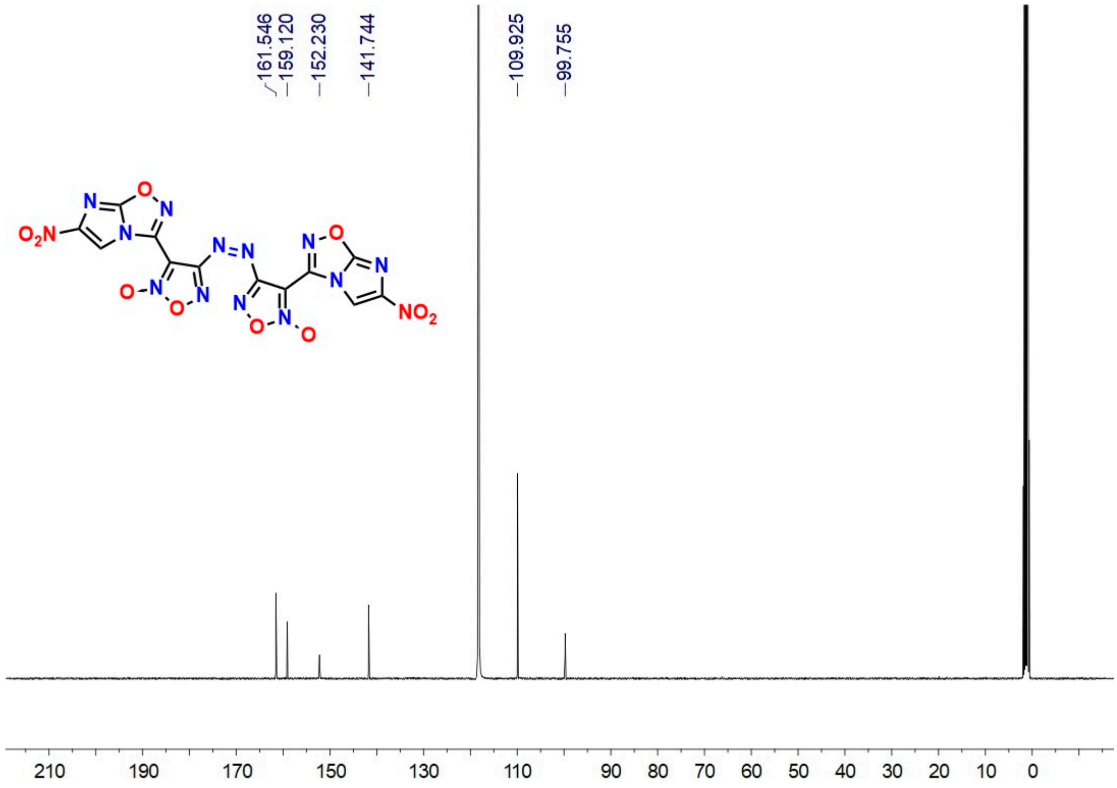

Figure S12. ^{13}C NMR Spectrum of **8** (CD_3CN, 100 MHz)

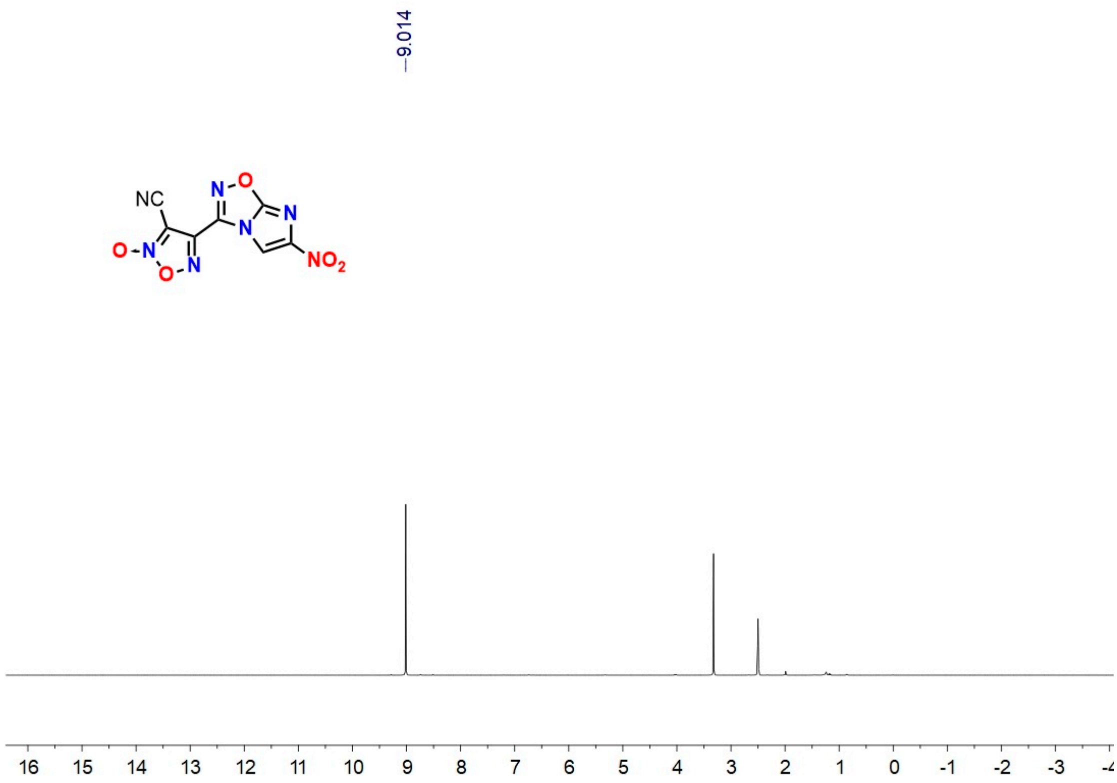

Figure S13. ^{1}H NMR Spectrum of **10** (DMSO, 400 MHz)

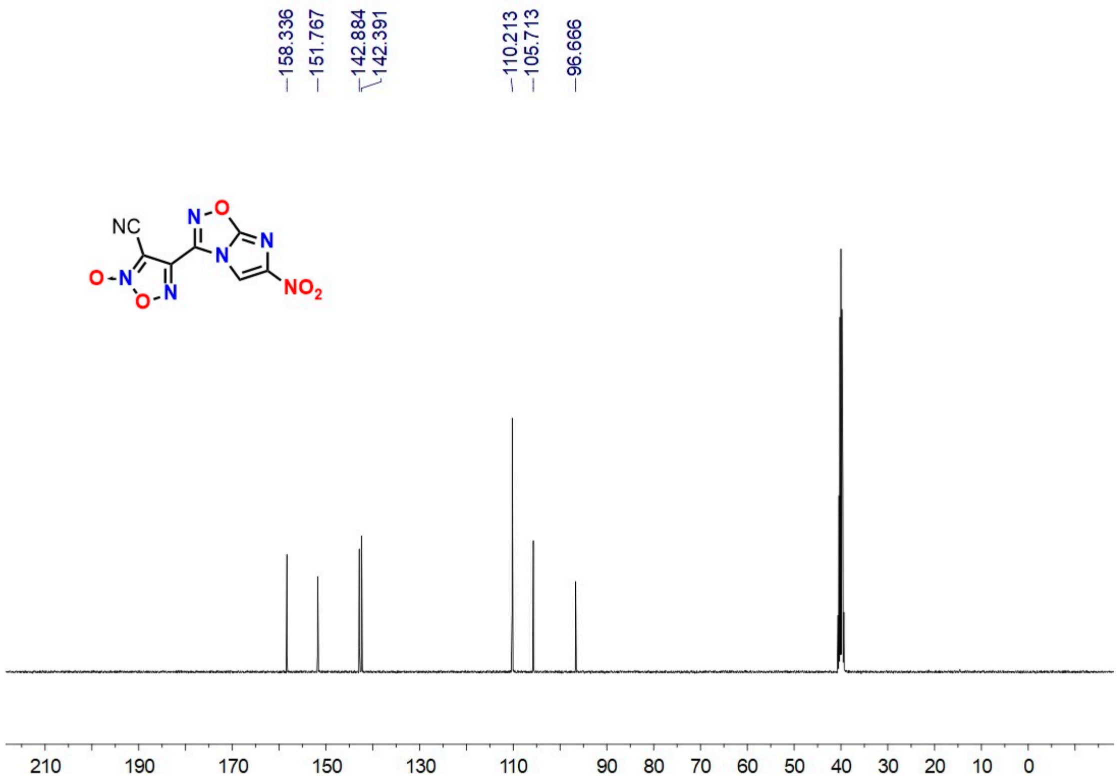

Figure S14. ^{13}C NMR Spectrum of **10** (DMSO, 100 MHz)

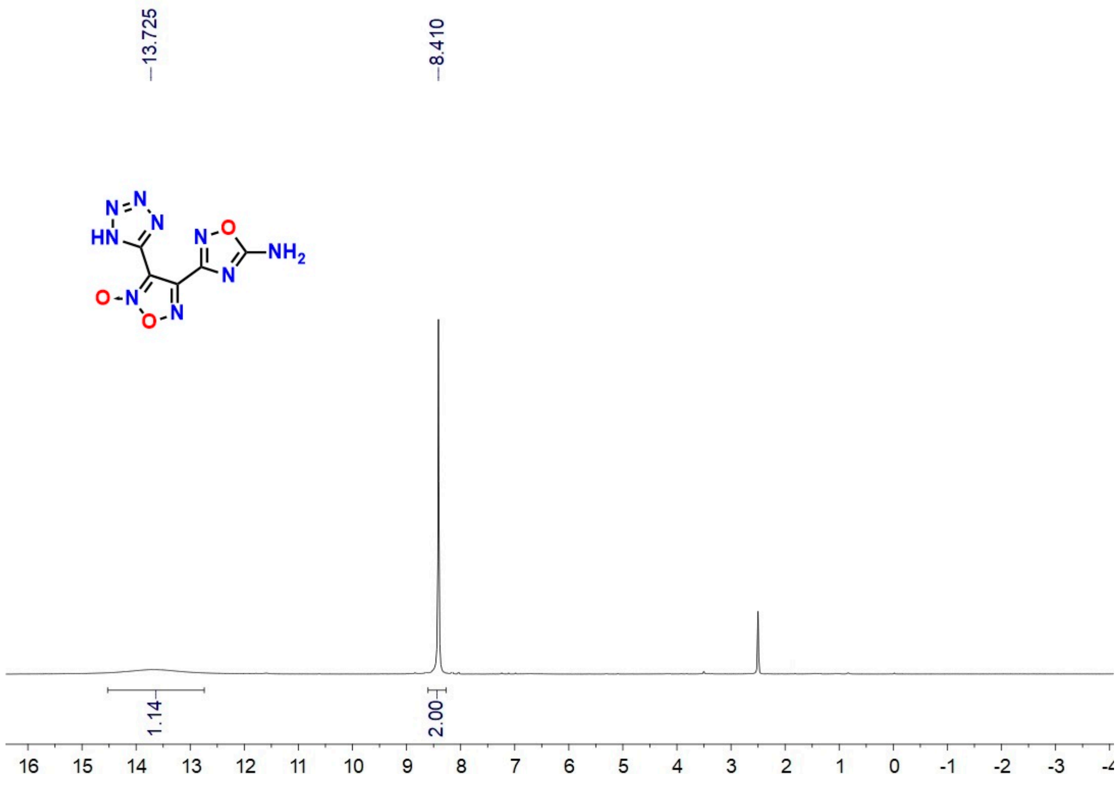

Figure S15. ^{1}H NMR Spectrum of **11** (DMSO, 400 MHz)

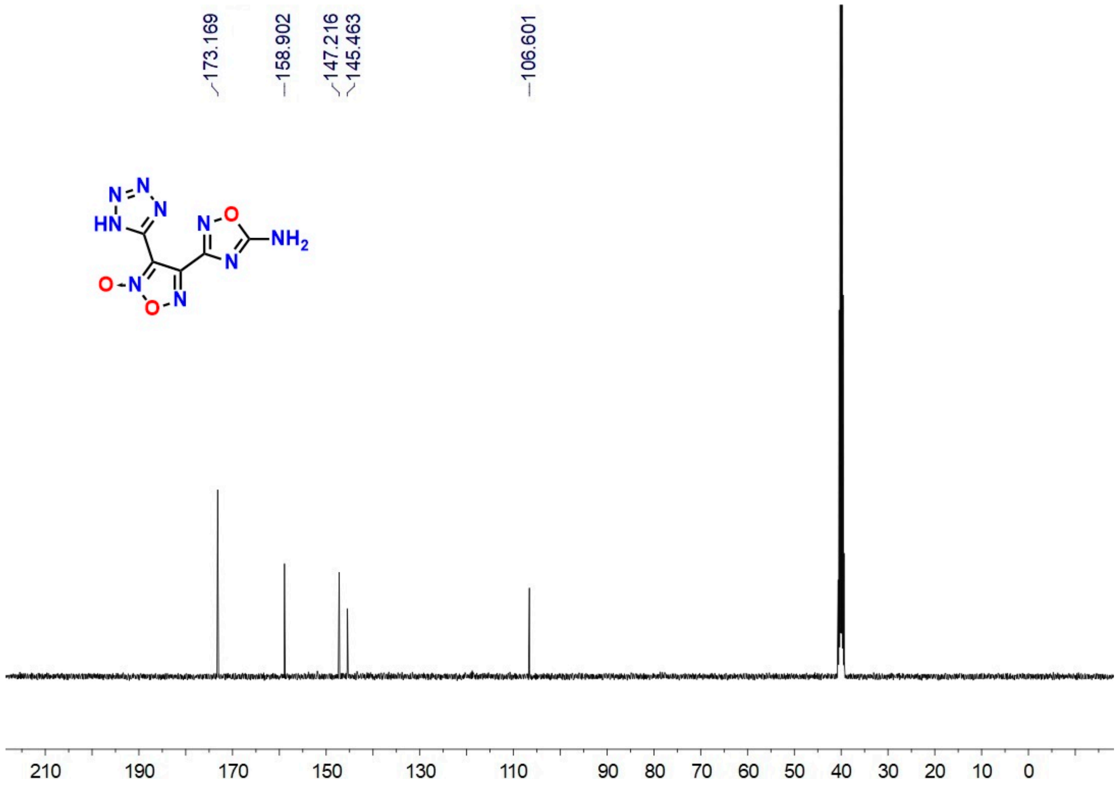

Figure S16. ^{13}C NMR Spectrum of **11** (DMSO, 100 MHz)

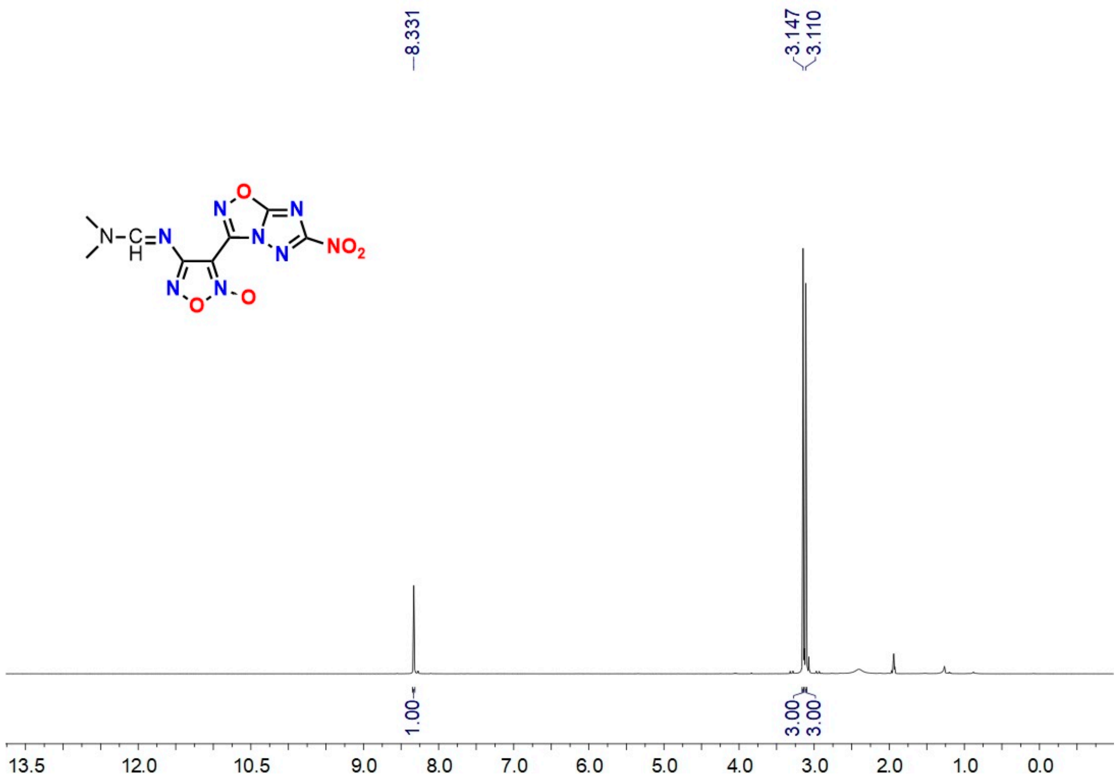

Figure S17. ^{1}H NMR Spectrum of **5a** (CD_3CN, 400 MHz)

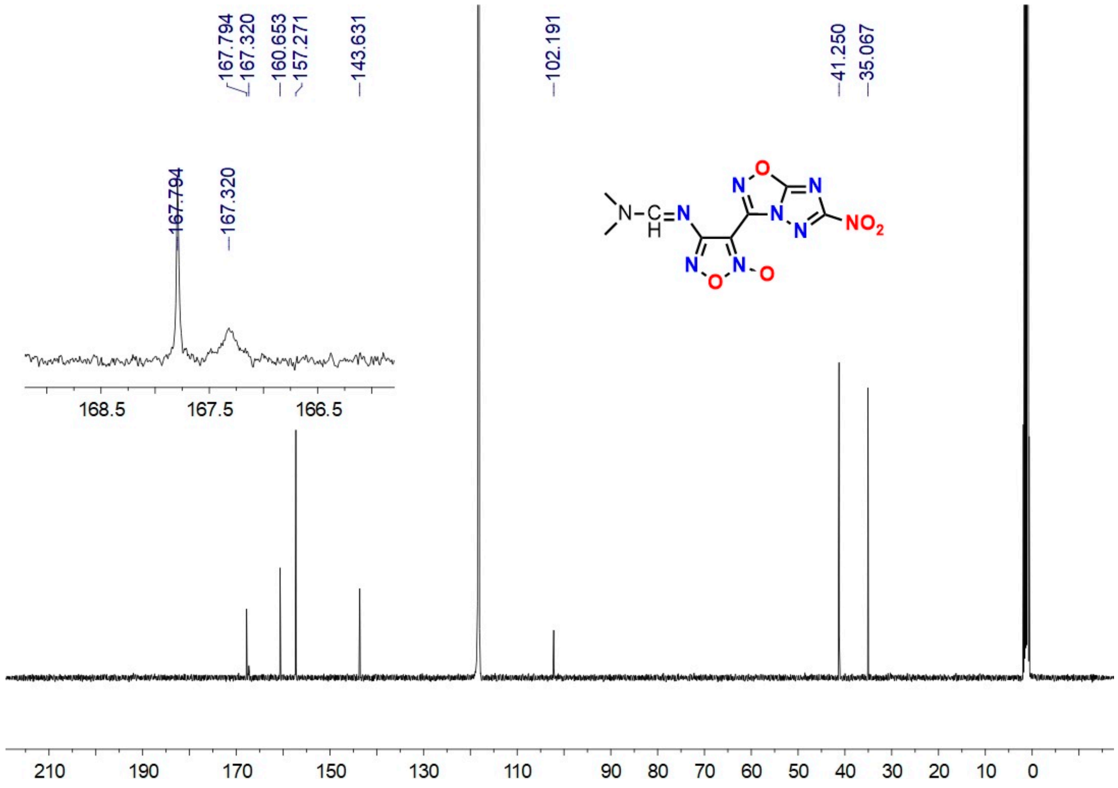

Figure S18. ^{13}C NMR Spectrum of **5a** (CD_3CN, 100 MHz)

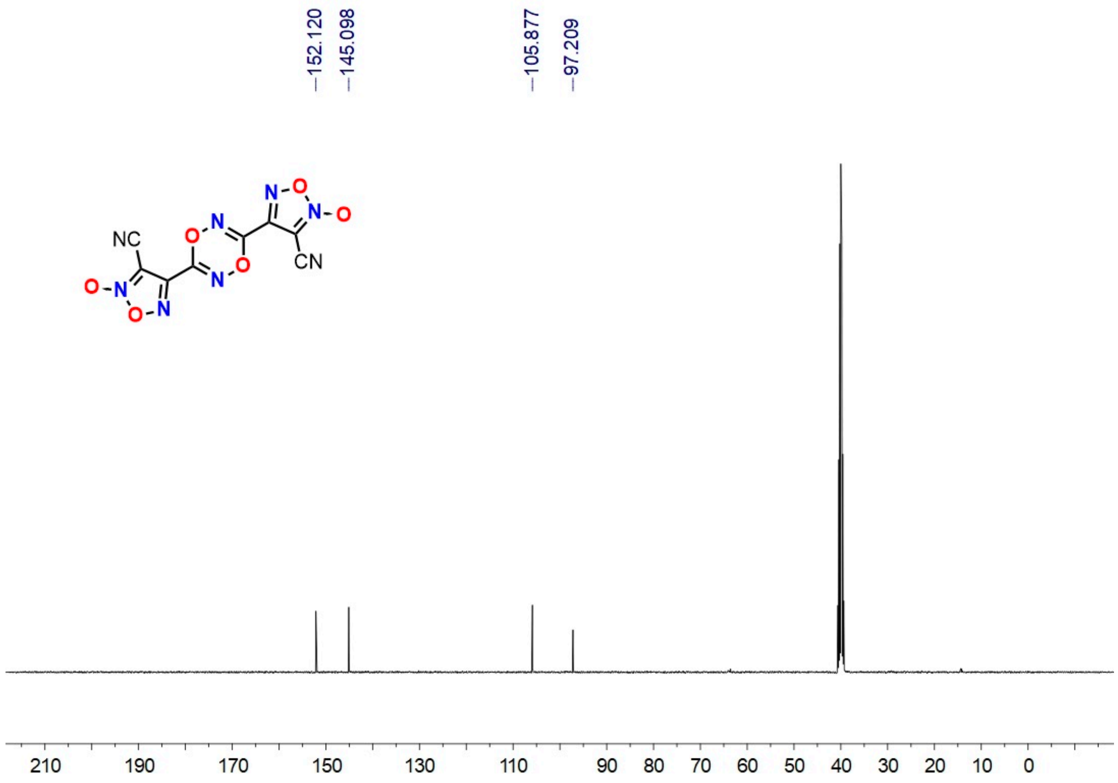

Figure S19. ^{13}C NMR Spectrum of **12** (DMSO, 100 MHz)

IV. DSC curves

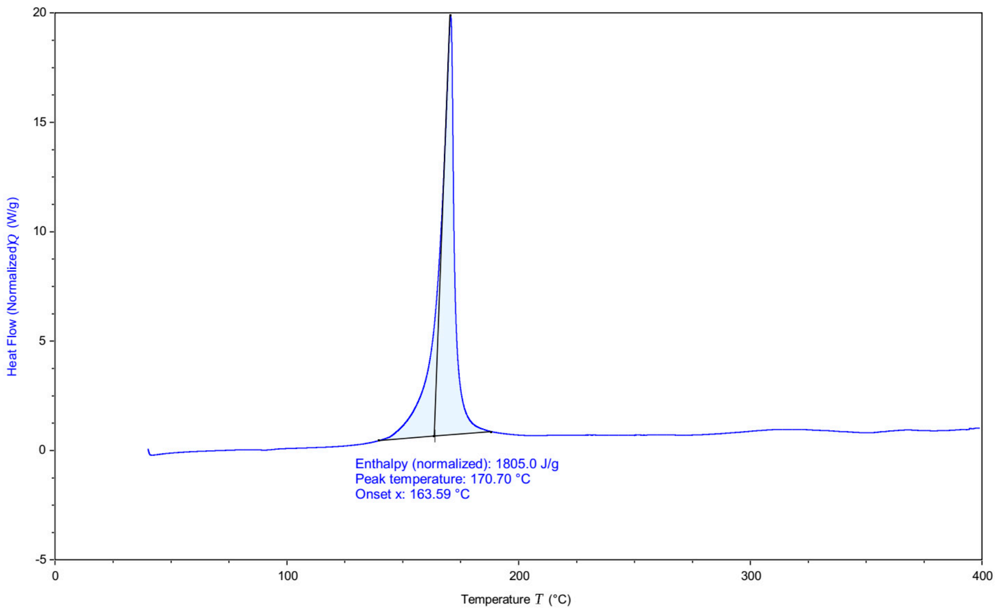

Figure S20. DSC curve of compound **6** at 5 °C min^{-1}

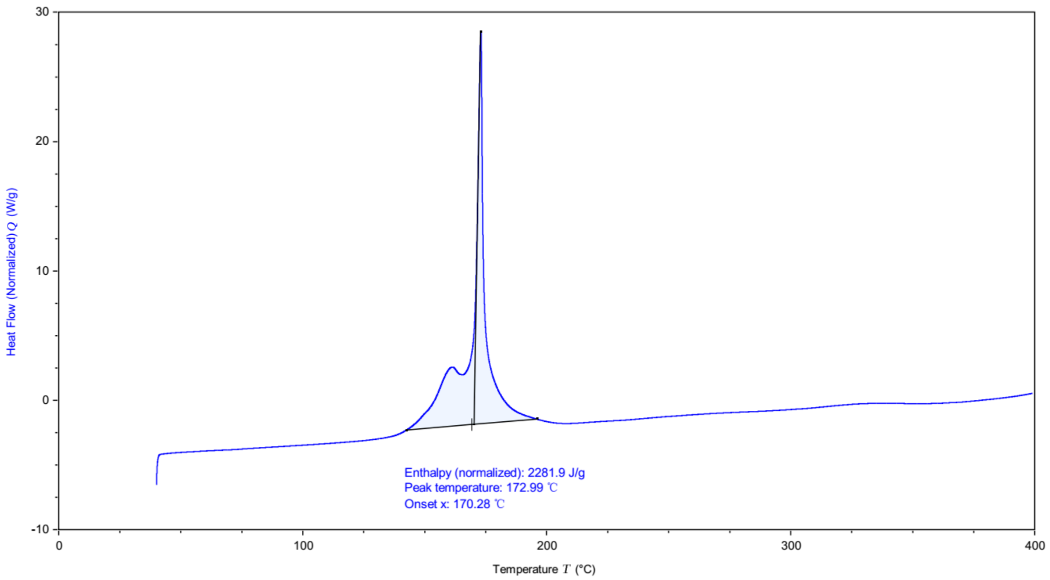

Figure S21. DSC curve of compound **7** at 5 °C min^{-1}

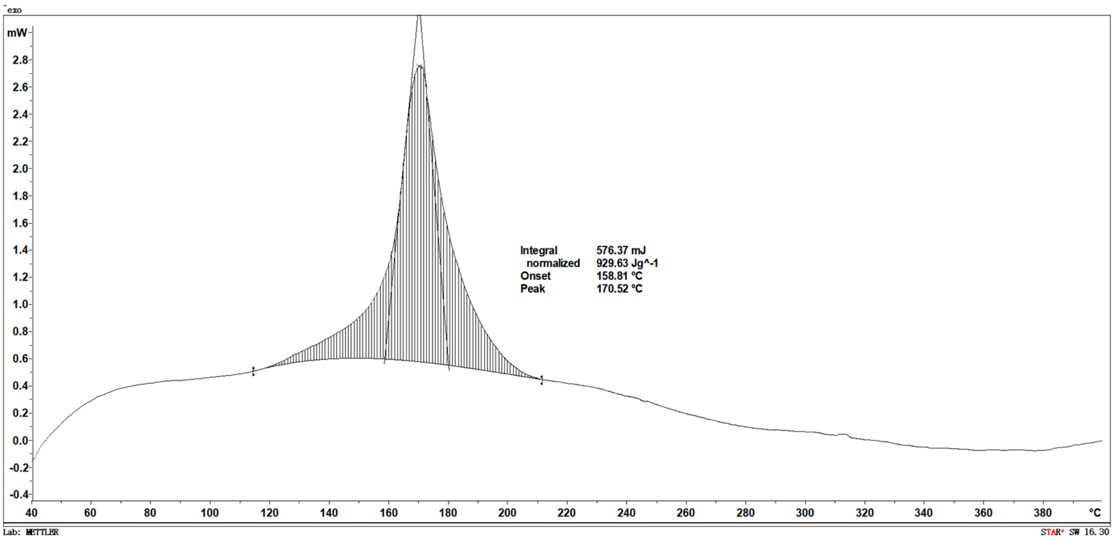

Figure S22. DSC curve of compound **8** at 5 °C min^{-1}

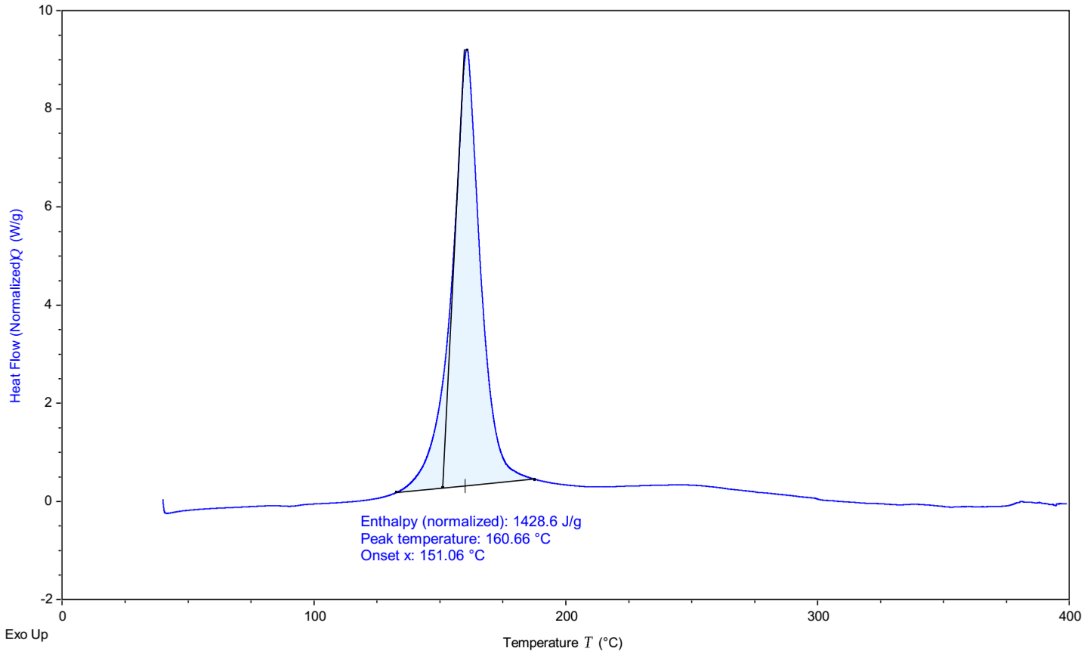

Figure S23. DSC curve of compound **5** at 5 °C min^{-1}

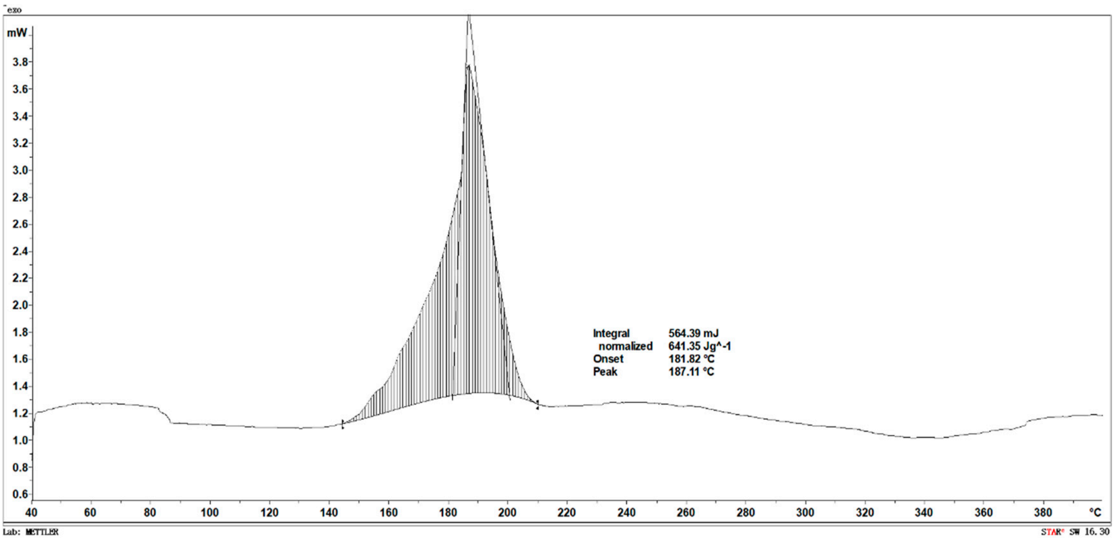

Figure S24. DSC curve of compound **10** at 5 °C min^{-1}

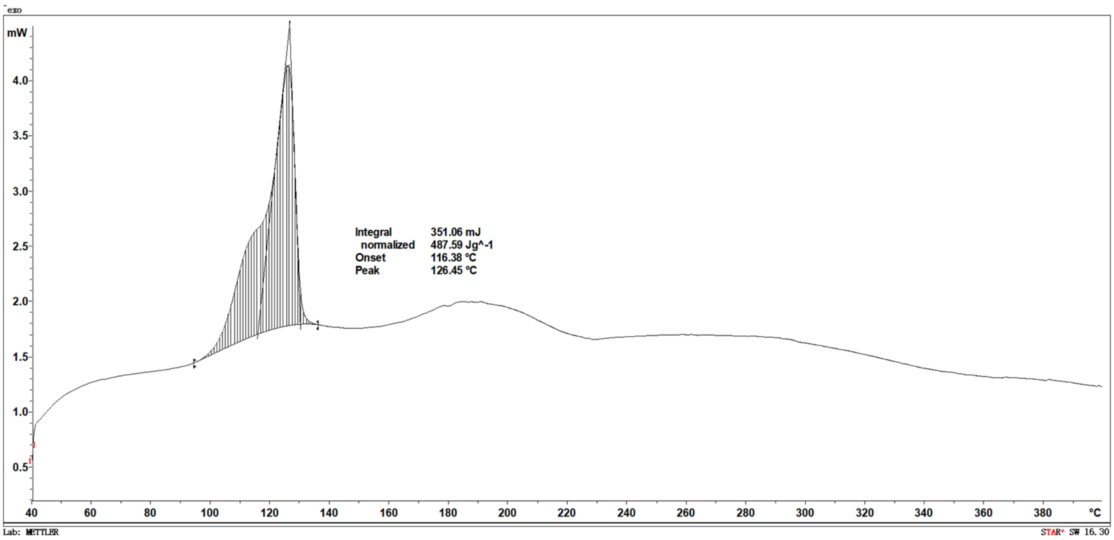

Figure S25. DSC curve of compound **5a** at 5 °C min^{-1}

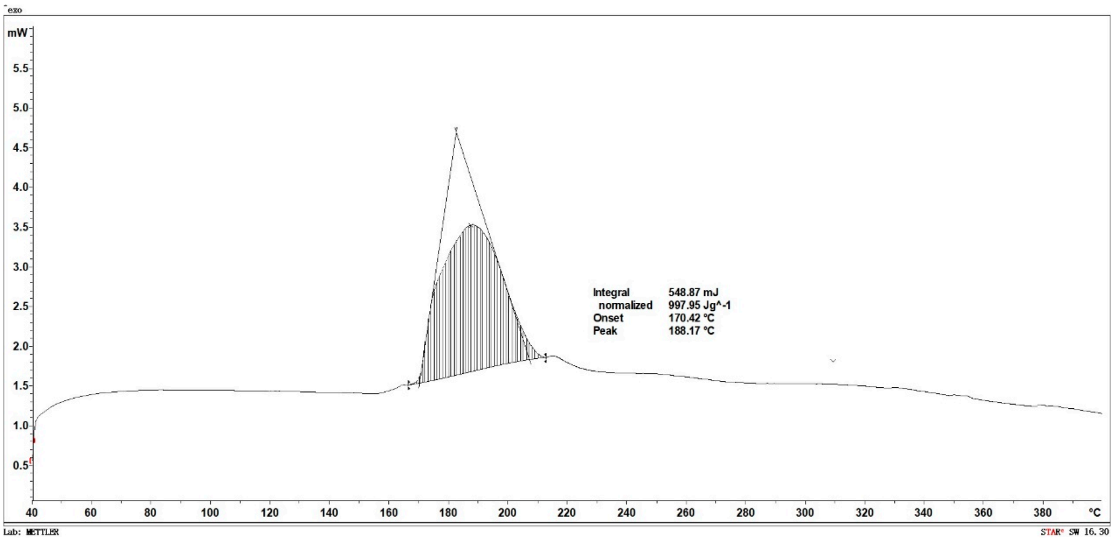

Figure S26. DSC curve of compound **12** at 5 °C min^{-1}
